# Greenstone burial–exhumation cycles at the late Archean transition to plate tectonics

**DOI:** 10.1038/s41467-022-35208-2

**Published:** 2022-12-22

**Authors:** Zibra Ivan, Kemp Anthony I S, Smithies R Hugh, Rubatto Daniela, Korhonen Fawna, Hammerli Johannes, Johnson Tim E, Gessner Klaus, Weinberg Roberto F, Vervoort Jeff D, Martin Laure, Romano Sandra S

**Affiliations:** 1 Geological Survey of Western Australia, 100 Plain Street, 6004 East Perth, WA Australia; 2 School of Earth, Atmosphere and Environment, Monash University, Clayton, VIC Australia; 3 School of Earth Sciences, University of Western Australia, Perth, 6009 Australia; 4 School of Earth and Planetary Sciences, the Institute for Geoscience Research (TIGeR), Timescales of Mineral Systems group, Curtin University, Bentley, Australia; 5 Institute of Geological Sciences, University of Bern, 3012 Bern, Switzerland; 6 Institut des Sciences de la Terre, University of Lausanne, 1015 Lausanne, Switzerland; 7 School of the Environment Washington State University Pullman, Pullman, WA 99164-2812 USA; 8 Centre for Microscopy, Characterisation and Analysis, the University of Western Australia, Perth, WA 6009 Australia

**Keywords:** Precambrian geology, Geochemistry, Tectonics, Petrology, Geodynamics

## Abstract

Converging lines of evidence suggest that, during the late Archean, Earth completed its transition from a stagnant-lid to a plate tectonics regime, although how and when this transition occurred is debated. The geological record indicates that some form of subduction, a key component of plate tectonics—has operated since the Mesoarchean, even though the tectonic style and timescales of burial and exhumation cycles within ancient convergent margins are poorly constrained. Here, we present a Neoarchean pressure–temperature–time (*P–T–t*) path from supracrustal rocks of the transpressional Yilgarn orogen (Western Australia), which documents how sea-floor-altered rocks underwent deep burial then exhumation during shortening that was unrelated to the episode of burial. Archean subduction, even if generally short-lived, was capable of producing eclogites along converging lithosphere boundaries, although exhumation processes in those environments were likely less efficient than today, such that return of high-pressure rocks to the surface was rare.

## Abstract (web summary)

Supracrustal rocks of the Yilgarn orogen underwent deep burial, and later syn-shortening exhumation. Archean exhumation processes were likely less efficient that today, so that complete exhumation of high-pressure rocks was rare and accidental.

## Introduction

Understanding which geodynamic mode(s) prevailed on our planet before plate tectonics is a major challenge in Earth science^1^. The secular evolution in global trends of geochemical^2,3^, metamorphic^4,5^, geophysical^6^ and paleomagnetic^7^ data from Archean rocks suggest that, towards the end of the Archean Eon (3200–2500 Ma), Earth underwent a fundamental secular shift between geodynamic modes^8^. This shift has been widely interpreted to represent the transition from a stagnant lid convection regime^9^, dominated by mantle upwelling and episodic, local, short-lived subduction events^3,9–11^, to some form of plate tectonics (the archetype of a mobile-lid tectonic regime) that likely was complete by the early Paleoproterozoic^12^. Nevertheless, the proposed timing for the onset of plate tectonics remains uncertain, encompassing most of Earth’s history^13^. To complicate the picture further, such a transition may have taken place via the transient establishment of other ancient forms of mobile lid behaviour that may have differed from modern plate tectonics^14^. Plate tectonics is characterized by rigid plates bounded by an interconnected network of plate boundaries comprising mid-ocean ridges, subduction zones and transform faults^13^. Therefore, the main challenge when interrogating the fragmented Archean rock record is to establish whether a given geological feature was part of a global network.

Structural studies represent an important but often overlooked tool to improve our understanding of early-Earth tectonic processes and geodynamic environments^15^. Two end-member styles of Archean crustal deformation are generally termed “dome-and-keel” and “linear imbricated belts”^8,16^ that may reflect distinct geodynamic environments^17^. Dome-and-keel domains were shaped by the juxtaposition of rising granite batholiths and sinking denser greenstones, with a tectonic style dominated by gravitational instabilities and body forces^18^. In these environments, cycles of deep-burial and exhumation of supracrustal rocks may have occurred in a few Myr only^19^, during periods of tectonic quiescence and/or extension, as in the case of the East Pilbara terrane^18^ and the Meso- to Neoarchean pre-orogenic evolution of the Yilgarn Craton (Western Australia)^20^. The dome-and-keel tectonic style lacks clear modern analogues, thereby offering limited information about how Earth transitioned to plate tectonics. In contrast, linear imbricated belts are generally interpreted to reflect accretionary processes associated with production of juvenile crust along ancient crustal boundaries^3,16,21^, and are characterized by large-scale shear zones juxtaposing crustal blocks with contrasting evolutionary histories. Archetypal examples of this tectonic style are the Superior Province (Canada) and the western Yilgarn Craton, both of which are characterized by large-scale transpressional shear zones^22,23^, and greenstones exhibiting “arc-like” geochemical signatures, interpreted to reflect subduction-accretion processes^10,24^. Furthermore, both cratons contain seismic reflectors in the uppermost mantle, interpreted to reflect relict suture zones associated with Archean subduction^25,26^. These features, together with overall crustal architecture^6^, stratigraphic and paleomagnetic data^8^, have been taken as evidence for the existence of accretionary orogens—and therefore of some local form of subduction—since at least the Mesoarchean^27^, although discordant views exist^28,29^.

One way to increase our understanding of the modality and emergence of plate tectonics is by examining the structural and metamorphic record of rocks exposed along major tectonic boundaries within Archean terranes characterized by linear imbricated belts. These are the most likely environments in which plate tectonic indicators can be found on ancient continents^30^. Although the metamorphic record preserved in these belts shows that of rocks equilibrated at depths of up to ~50 km during the Archean^5^, consistent with some form of convergent tectonic regime, both the tectonic style and timescales of burial–exhumation cycles remain poorly constrained^31–34^. To overcome these issues, here we integrate geochemical, geochronological and metamorphic data with available structural data^35^, to unravel the *P–T–t* paths of Archean greenstone belts.

We document a complete cycle of deep burial and exhumation in metamorphosed volcano-sedimentary rocks of the Waroonga Greenstone Belt (WGB) along the Ida Fault, a major tectonic boundary (≥500 km long) within the Yilgarn Craton.

**Fig. 1:**
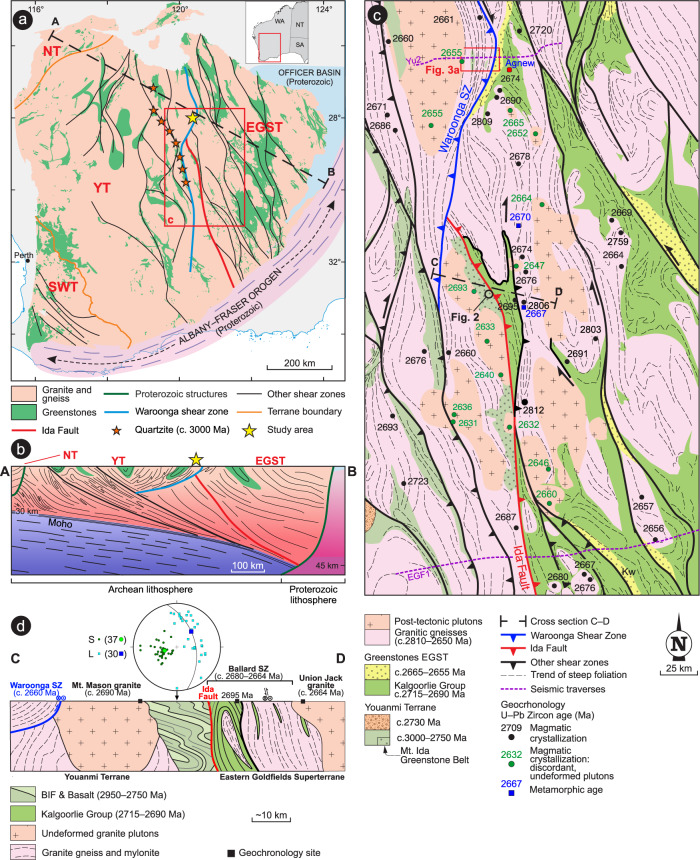
Geological maps and cross sections, Yilgarn Craton. **a** Simplified geological map of the Yilgarn Craton, showing the subdivision into main terranes (NT: Narryer; YT: Youanmi; SWT: Southwest; EGST: Eastern Goldfields Superterrane), and the network of craton-scale shear zones. The black dashed line shows the trace of the cross section shown in **b**. **b** Geological cross section across the northern part of the craton, illustrating the main crustal architecture, characterized by east-dipping Moho and listric, crustal-scale shear zones. **c** Geological map of the central portion of the Yilgarn Craton. Note that the Ida Fault is truncated by the Waroonga Shear Zone. Ages of magmatic crystallization for granite plutons are from Geoscience Australia and Geological Survey of Western Australia databases. **d** Geological cross-section across the central portion of the Ida Fault. The equal-angle plot shows poles to gently east-dipping foliation and the gently east-plunging stretching lineation for the banded iron formation (BIF)-basalt sequence (with indication of mean values), in striking contrast with that of the transpressional fabric exposed in the rest of the craton, which is invariably steeply dipping^23^. The gently dipping fabric is postdated by the undeformed, c. 2690 Ma Mt. Mason granite, which was emplaced at depth, during the deposition of the Kalgoorlie Group. **c** for cross-section trace. Compilation of maps and cross-sections is based on combined field and geophysical data.

The Yilgarn Craton mostly exposes Meso- to Neoarchean granite–greenstone terranes (Fig. 1) that were largely unaffected by post-Archean deformation^36^. The oldest-preserved supracrustal rocks are fluvial to shallow-marine quartzites deposited between 3250 and 2935 Ma^37^ that were deposited during rifting of the Eoarchean Yilgarn proto-craton^38^. Quartzites are exposed along a ~330 km-long belt (Fig. 1a), indicating a craton-scale rifting event. Subsequently, in the period 2960–2750 Ma, craton-wide mafic to ultramafic sequences interlayered with banded iron formation (BIF) developed in deep-marine basins during prevailing lithospheric extension^39,40^. Large-scale horizontal shortening started after the deposition of the 2750 Ma Wilgie Mia Formation, at c. 2730 Ma^40^. The arc-like geochemical signature of the <2820 Ma component of the greenstone sequence is interpreted to reflect a subduction-related genesis^41^ and, at c. 2730 Ma, collision between the Narryer Terrane to the west with the rest of the Yilgarn Craton^26,38^. This collision was the likely driver for the onset of the 2730–2650 Ma Neoarchean Yilgarn Orogeny^40^, which produced a transpressional belt characterized by crustal-scale shearing and syntectonic granitic magmatism^23^. In the central–eastern part of the craton (i.e., Eastern Goldfields Superterrane, EGST, Fig. 1), a younger, craton‐scale (~800 km along strike) cycle of asthenosphere-derived magmatism produced a > 10‐km‐thick sequence of mafic–ultramafic lavas and intrusive rocks (Kalgoorlie Group), during a 2715–2690 Ma rifting along the eastern flank of the Ida Fault^10^. Horizontal shortening resumed at c. 2680 Ma^23^, during which metamorphosed greenstones of the WGB, which record an earlier metamorphic peak at ~13 kbar^35^, were exhumed along the c. 2660 Ma Waroonga shear zone (WSZ)^35^. The age of this shearing event is constrained by the c. 2662 Ma depositional age of the footwall sedimentary sequence, and by the c. 2655 Ma crystallization age of granites intruded into the shear zone^35^. The lithospheric wedge produced during the orogeny includes a network of east dipping, crustal-scale shear zones, concordant with a regionally east-dipping Moho^6^ (Fig. 1b). The minimum age of this lithospheric architecture is c.2730 Ma^40^.

As part of this study, we investigated an area along the Ida Fault^42^ at the boundary between the Youanmi Terrane and the EGST, in the central Yilgarn Craton (Fig. 1). The two terranes differ in terms of greenstone stratigraphy and tectonic evolution^38^.

**Fig. 2:**
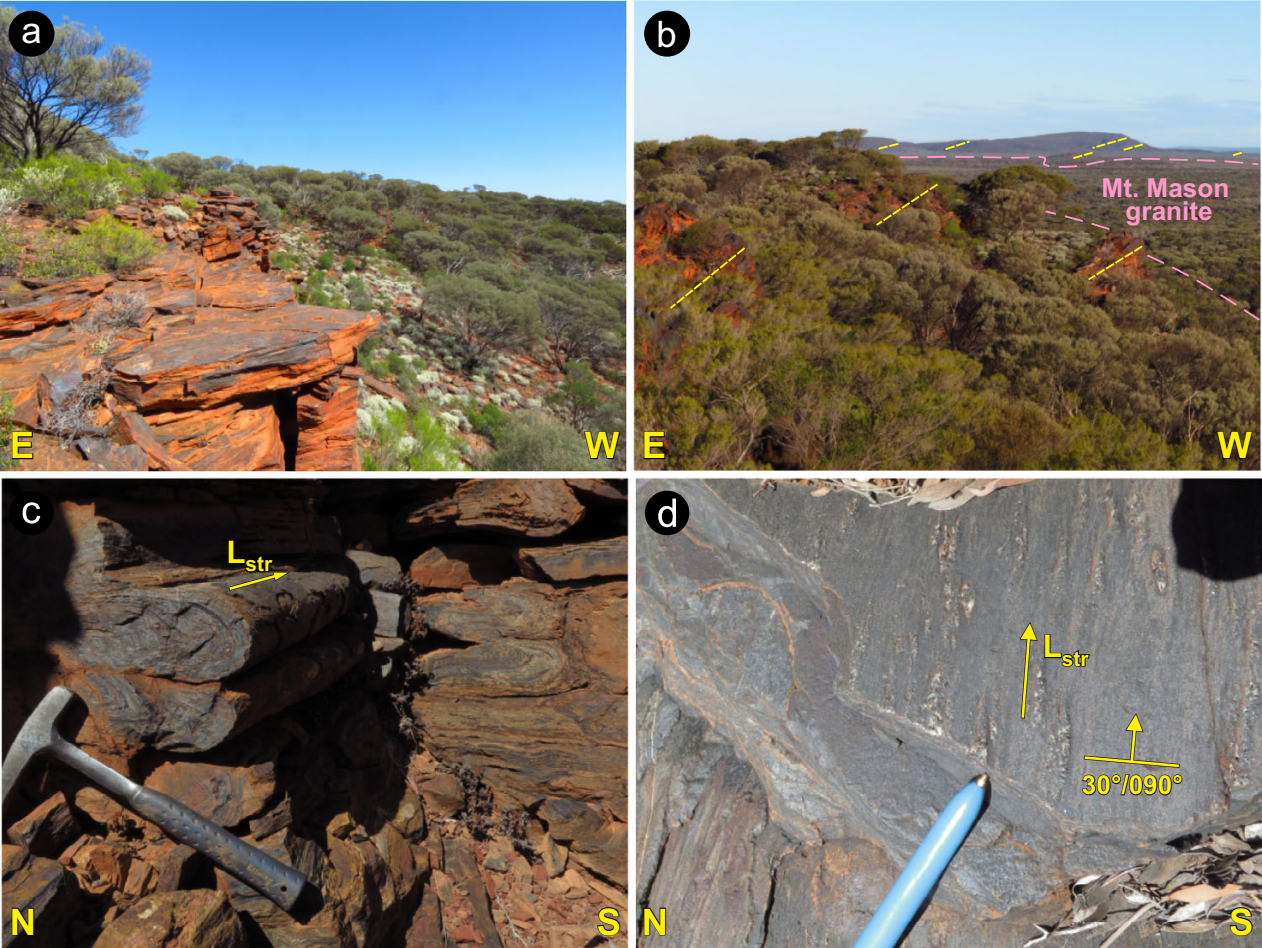
Prominent outcrop-scale features of the Mount Ida greenstone belt. Outcrops location shown in Fig. 1b. **a** Approximately 20 m-thick BIF (Banded Iron Formation) ridge, showing gently east-dipping transposed bedding. **b** Panoramic view from Mt Mason, looking southward. Interlayered, weathering-resistant BIF and basalts are associated with a prominent topographic scarp, 100–200 m higher than the flat landscape at west, dominated by the more erodible granite. The consistently east dipping transposed bedding in BIF (marked by the dashed lines) can be followed for nearly 200 km along strike. **c** Close-up view of the transposed bedding in BIF, with tight isoclinal folds whose axes, subparallel to the stretching lineation, are invariably gently east-plunging. **d** View from above (parallel to the transposed bedding) of BIF exposure. The prominent stretching lineation is marked by elongate quartz-magnetite aggregates.

The cross-section across the central portion of the Ida Fault (Fig. 1c, d) offers a synthesis of the regional-scale architecture, and of the time constraints for the tectonic evolution of the area. The main tectonic fabrics along this major crustal boundary developed during regional-scale D_1_ and D_2_ events of transpressional, synmagmatic shearing along the east-dipping, c. 2680–2664 Ma Ballard shear zone^23^ and the west-dipping, c. 2660 Ma WSZ^35^. These two events postdate the prominent c. 2730 Ma^23^ regional-scale low-angle tectonic fabric, which is well preserved for ~180 km along strike (Fig. 2) of the ≥2750 Ma BIF-basalt sequence of the Mt Ida greenstone belt, along the western flank of the Ida Fault (Fig. 1c and d).

**Fig. 3:**
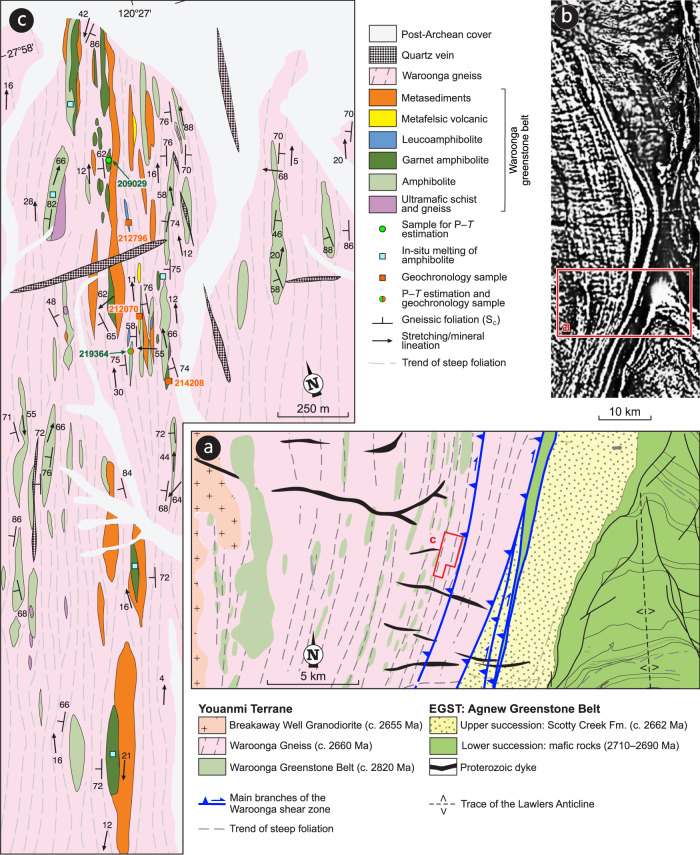
Geological maps of the Waroonga shear zone. **a** Geological map of the study area, across the central portion of the Waroonga shear zone (Fig. 1c for location). The rectangle shows the location of the map shown in panel **c**. **b** Merged magnetic anomaly map of the Waroonga shear zone, as part of the magnetic anomaly grid (40 m) of Western Australia, Version 1 – 2013. The full map is available on the Geological Survey of Western Australia (GSWA) website: http://www.dmp.wa.gov.au/Geological-Survey/Regional-geophysical-survey-data-1392.aspx. The rectangle shows the location of panel **a**. **c** Geological map of the sampling area, showing location of samples collected for geochronology and *P–T* estimations.

The WGB is a metamorphosed and tectonically attenuated volcano-sedimentary sequence exposed in the hanging wall of the WSZ. Our work is focused on the central part of the shear zone, along a nearly continuous ~15 km-long east–west transect (Fig. 1b). The Waroonga shear zone consists of the Waroonga Gneiss, a syntectonic, c. 2660 Ma tonalitic–granitic pluton that contains 10–1000 m long slivers of WGB greenstones (Fig. 3). The WGB mainly contains amphibolite (metabasalt) and metamorphosed BIF, with subordinate metamorphosed felsic volcanic rocks, mica schist, quartzite, leucoamphibolite and ultramafic schist. The greenstone slivers in the Waroonga gneiss have high aspect ratios (10:1 to 100:1) in map view, with long axes aligned subparallel to the roughly north-striking, arcuate pattern of the Waroonga shear zone (Fig. 3). Amphibolite from the WGB shows variably retrogressed high-pressure granulite-facies assemblages^35^. Retrogression in the leucoamphibolite included the development of a migmatitic fabric (Supplementary section 3). Most greenstone slivers preserve internal lithological contacts interpreted to represent transposed primary contacts within the original volcano-sedimentary succession^35^.

**Fig. 4:**
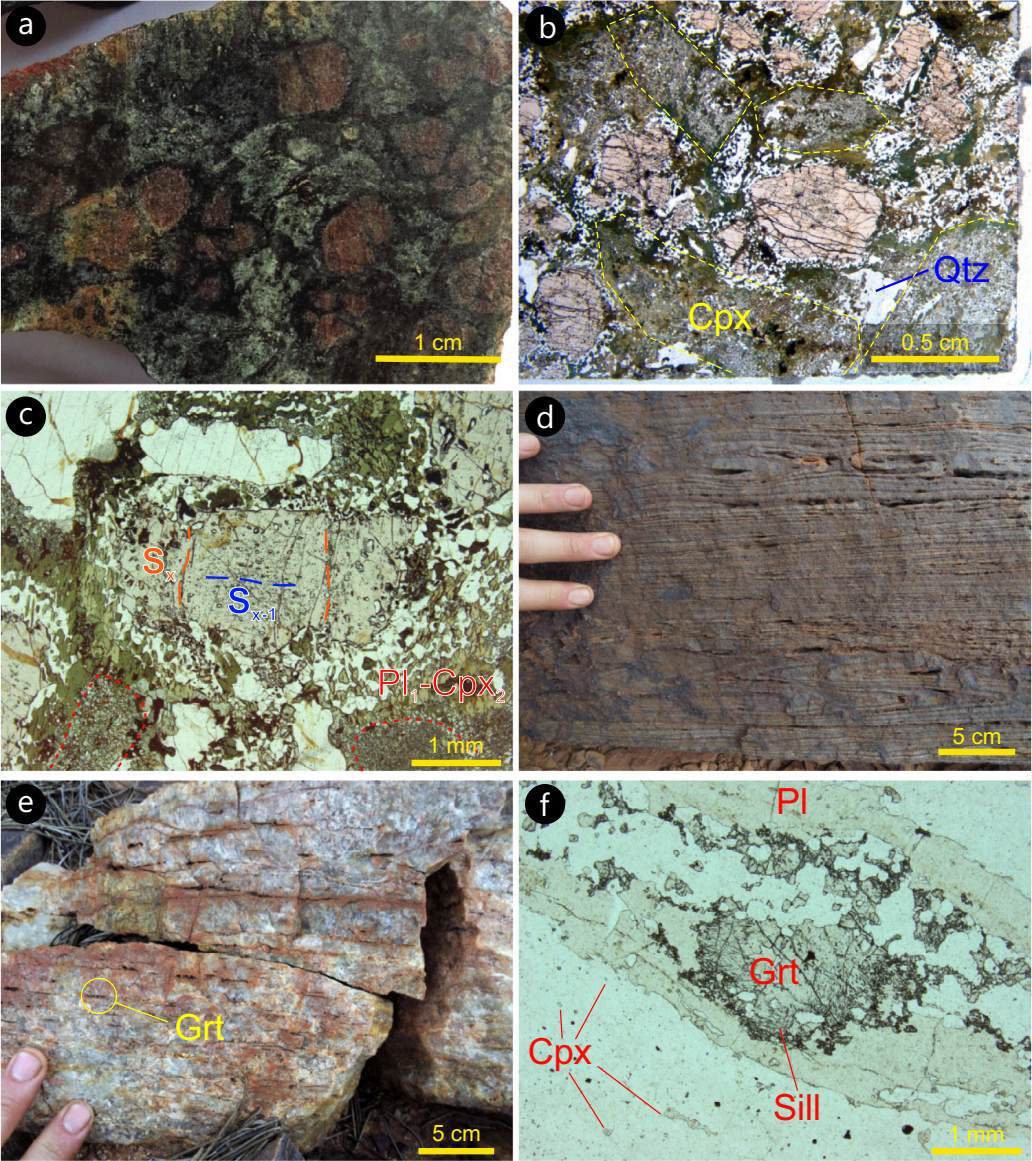
Typical meso- and microscale features of the main rock types in the Waroonga greenstone belt. **a** Polished hand specimen (sample 209029) from the core of a garnet amphibolite body. Subhedral to euhedral, cm-sized garnet and pale-green clinopyroxene porphyroblasts (M1 assemblage) show variable degrees of retrogression, as marked by hornblende-rich black rims. **b** Plane polarized light view of hand sample shown in **a**, highlighting the texturally preserved peak (M1) assemblage of clinopyroxene, garnet, quartz and rutile. Peak pyroxene (Cpx1), whose outline is marked by dashed yellow lines, is replaced by clinopyroxene (Cpx2)–plagioclase1 symplectites (M2 assemblage) and by hornblende–plagioclase2 symplectites. The latter developed at the expenses of both Cpx1 and garnet. **c** Garnet porphyroblast (sample 209029) preserving evidence of two internal foliations (S_x_ crenulating S_x-1_ foliation) that, since they predate garnet growth, find no equivalence in mesoscale fabrics observable in the field. **d** Transposed bedding in strongly deformed Banded Iron Formation (BIF), showing rootless, isoclinal folds. **e** Transposed bedding in strongly deformed quartzite, in primary contact with BIF. **f** Micrograph from quartzite shown in **e**, with granulite-facies assemblage comprising skeletal garnet (with sillimanite inclusions) wrapped by plagioclase ribbons, and small clinopyroxene porphyroblasts scattered in quartz ribbons. Plane polarized light.

Garnet amphibolite is medium- to coarse-grained (average grain size 0.5 − 2 cm, Fig. 4a) with euhedral to anhedral garnet and pyroxene porphyroblasts that are surrounded by amphibole-rich symplectites (compare Fig. 4a, b). It contains a clinopyroxene–garnet–orthopyroxene–hornblende–plagioclase–quartz –rutile assemblage. Inclusion trails in garnet porphyroblasts define a crenulation cleavage (Fig. 4c), indicating that final porphyroblast growth at peak metamorphic conditions postdated at least two generations of tectonic foliation. Leucoamphibolite is fine-grained and preserves meso-and microstructures indicative of a partial melting event that predated c. 2660 Ma exhumation along the WSZ^35^; these microstructures are described in detail in the supplementary section 3.

Metamorphosed felsic volcanic rocks and mica schist contain quartz, muscovite, biotite, plagioclase, K-feldspar and sillimanite. These felsic rocks retain primary contacts with BIF, amphibolite and ultramafic rocks, within the same, composite greenstone slivers, demonstrating the volcano-sedimentary nature of the WGB. Bedding in BIF shows a variable degree of transposition, with thickening in fold hinges and marked attenuation along fold limbs (Figs. 2c and 4d). BIF layers are commonly coarse-grained and granoblastic. Most BIF layers are enriched in acicular amphibole that may contain cores of clinopyroxene. BIF is associated with layers of coarse-grained quartzite (Fig. 4e), which contain elongate ribbons of garnet, quartz and plagioclase aggregates, including small, rounded clinopyroxene porphyroblasts. Garnet aggregates in places show a skeletal habit and include sillimanite needles (Fig. 4f). This assemblage suggests that, like mafic rocks, metasedimentary rocks of the WGB reached high-pressure granulite-facies conditions^43^. In the eastern part of the studied area, a group of greenstone slivers is clustered in an area 1.8 km across-strike by 3.7 km along strike (Fig. 3c). Pre- 2660 Ma high-grade assemblages of the WGB are best preserved in this area, which include the garnet amphibolite, leucoamphibolite, BIF and meta-felsic volcanic rocks studied here.

## Results

### Geochemistry and O isotopes in garnet

**Fig. 5:**
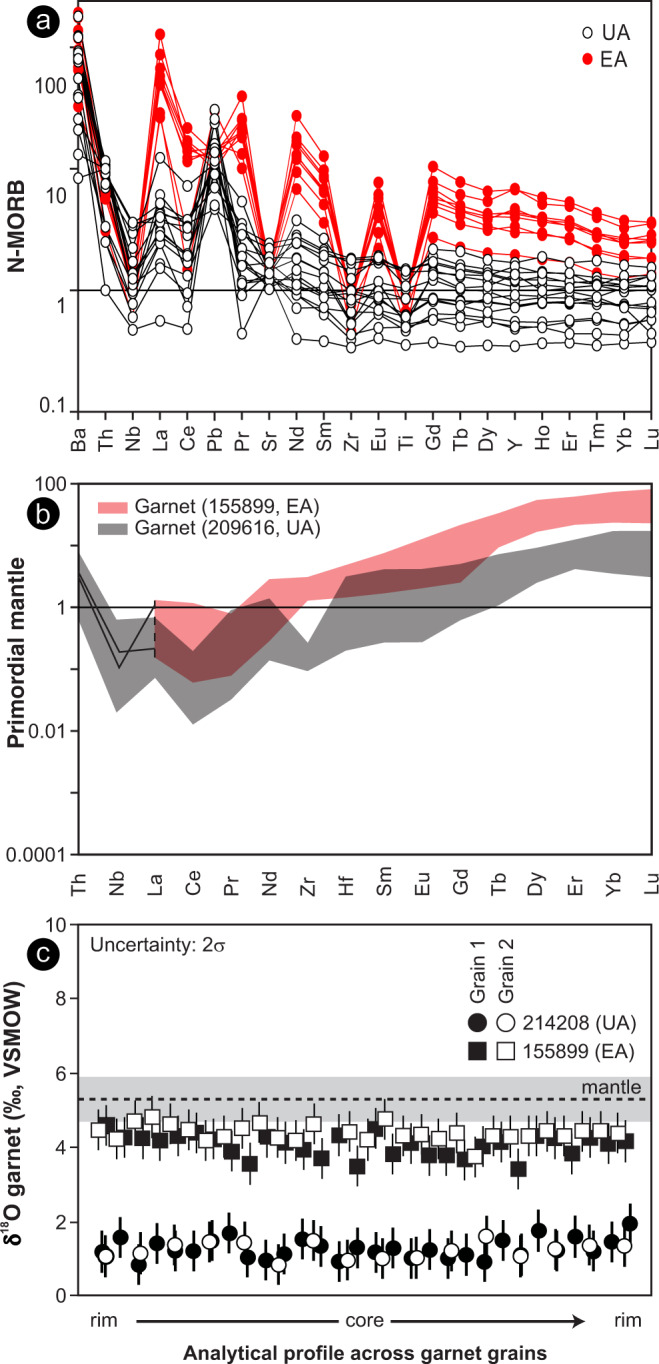
Trace-element and O-isotope compositions of amphibolite from the Waroonga greenstone belt. **a** Normal mid-ocean ridge basalts (N-MORB) normalized incompatible trace-element patterns of the garnet amphibolite, showing the distinctive differences in rare Earth elements (REE), large ion lithophile elements (LILE), Th and, in particular, high field strength elements (HFSE), in the EA group. **b** Mantle-normalised incompatible trace-element patterns of garnet in UA and EA. Compared with UA, EA garnets show strong enrichments in REE, without enrichment in Nb (only two analyses, both shown as traces, with Nb above detection) or Th. **c** Secondary ion mass spectrometer (SIMS) δ^18^O analyses across garnet grains of sample 214208 and 155899. The grey bar shows the mantle δ^18^O range^44^.

We determined the whole-rock major- and trace-element compositions of garnet amphibolite (*n* = 43) and BIF (*n* = 51) within the WGB to constrain the nature of the protoliths (Supplementary section 1). Amphibolite defines major-element trends typical of tholeiitic basalts of the Yilgarn Craton (Supplementary Figs 1 and 2) but, based on trace-element concentrations, includes distinct unenriched and enriched groups (UA and EA, respectively, Fig. 5a). The UA shows NMORB (normal mid-ocean ridge basalts)-like incompatible trace-element patterns, whereas the EA shows extreme enrichments in rare earth elements (REE) and Y (Fig. 5a). Notably, contrasts in the trace-element compositions of the two groups are not reflected in their mineralogy, textures or major-element geochemistry, although the trace element chemistry of garnet and hornblende in the UA and EA mimic those of their respective whole rocks (Fig. 5b and Supplementary Fig. 3a, b).

The close compositional similarity between UA and typical Yilgarn tholeiitic basalt (Supplementary Figs. 1 and 2), particularly in terms of melt-mobile elements such as light REE, Zr, Nb and Th, indicates that the compositions of the UA samples were largely unaffected by high-grade metamorphism. Based on similar compositional criteria, BIF from the WGB also falls into enriched and unenriched groups (Supplementary Fig. 3c).

Secondary ion mass spectrometer (SIMS) δ^18^O data show that garnet porphyroblasts within each sample are compositionally homogeneous (Fig. 5c). Garnet from typical EA rocks (sample 155899) has average δ^18^O values (vs. Vienna Standard Mean Ocean Water, VSMOW) of 4.2‰; ±0.6‰; (2σ), while those in typical UA examples (sample 214208) have distinctly lower δ^18^O values (average of 1.3‰; ±0.5‰;, 2σ), which are well below those of mantle rocks (~5.3‰; ±0.6‰;^44^) and among the lowest recorded δ^18^O values from garnet in mafic rocks^45^. Such low δ^18^O values in mafic rocks are typical of high-temperature seafloor alteration^46^ and are commonly preserved during metamorphism^47^.

### Zircon, monazite and garnet geochronology

**Fig. 6:**
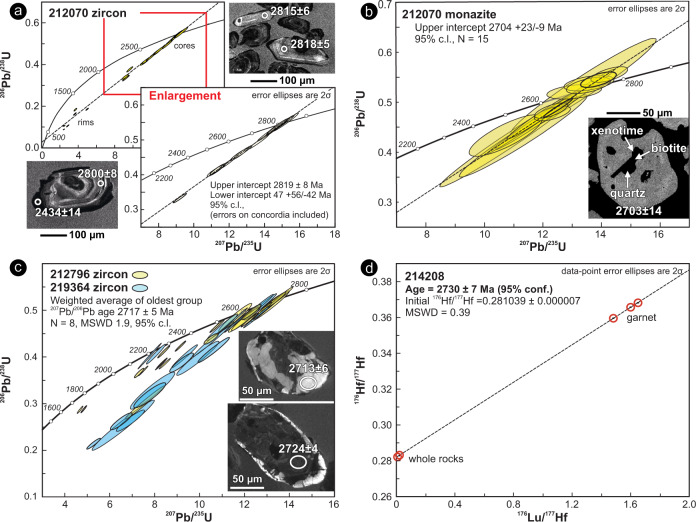
Geochronology results for zircon, monazite and garnet. **a**–**c** Concordia diagrams of U-Pb analyses of zircon and monazite. The inset in each panel shows representative cathodoluminescence (CL; zircon) and back-scattered electron (BSE; monazite) images of analysed grains with the spot location (white circle) and corresponding ^207^Pb/^206^Pb dates. **d** Lu–Hf isochron diagram showing data obtained from three garnet fractions, the corresponding whole rock, and a garnet-free whole rock aliquot. The red circles outline the position of individual data points, which are only barely resolvable at this scale.

To constrain the age of WGB deposition at the ocean floor, we separated zircon crystals from a metamorphosed felsic volcanic rock (sample 212070) that show core–rim microstructures (Fig. 6a). In cathodoluminescence (CL) images, the cores preserve oscillatory zoning and have high Th/U ratios (0.25–0.82) typical of magmatic growth, whereas the thin (<25 µm thick) rims are unzoned and generally have much lower Th/U (down to 0.02), typical of metamorphic zircon. Most core U–Pb analyses (12/15) yield the oldest ^207^Pb/^206^Pb dates and define a discordia with an upper intercept at 2819 ± 8 Ma (mean square weighted deviation, MSWD = 1.4; all ages reported at 95% confidence level) anchored by five concordant analyses (Fig. 6a and Tab. S10). The zoning, chemistry and consistent dates of the zircon suggest a magmatic origin. Given the occurrence of primary contacts between WGB lithologies, and the evidence of shared (i) alteration-related geochemical signature and (ii) metamorphic cycles (described below), we interpret the zircon core age to constrain the eruption age of the WGB at the ocean floor. Analyses of monazite from the same sample define a discordia with an upper intercept of 2704 + 23/−9 Ma (Fig. 6b). Eleven monazite analyses that are > 90% concordant define a ^207^Pb/^206^Pb age of 2702 ± 9 Ma (MSWD = 0.74).

We separated zircon crystals from leucoamphibolite (samples 212796 and 219364) to constrain the age of the migmatitic fabric. In CL, these crystals show complex mottled or chaotic zoning and contain abundant micro-inclusions (Fig. 6c); oscillatory zoning is rarely preserved. Concentrations of U and Th in CL-dark cores are high (415–1390 and 230–1900 ppm, respectively, with Th/U = 0.27–2.5), and the most extreme compositions correspond to discordant U–Pb analyses. ^207^Pb/^206^Pb dates range from 1917–2723 Ma in sample 212796, and 2230–2719 Ma in sample 219364 (Fig. 6c), with no systematic difference in dates between CL-dark domains and CL-bright rims. The microstructure and high U–Th contents of the zircon cores suggest intense alteration and metamictization. Although the analyses from both samples do not define a unique discordia, the oldest group of mostly concordant analyses has an average ^207^Pb/^206^Pb age of 2717 ± 5 Ma, which is taken to represent the timing of melt crystallization in the migmatite^48^ (Fig. 6c).

To constrain the age of peak metamorphism in the WGB, we performed Lu–Hf geochronology on a garnet amphibolite (sample 214208, UA). Analyses of two whole-rock (including a garnet-free matrix), and three different garnet fractions yielded a statistically significant isochron with a date of 2730 ± 7 Ma (Fig. 6d). We interpret this date to record garnet growth close to the metamorphic peak^49,50^.

### Metamorphic data

**Fig. 7:**
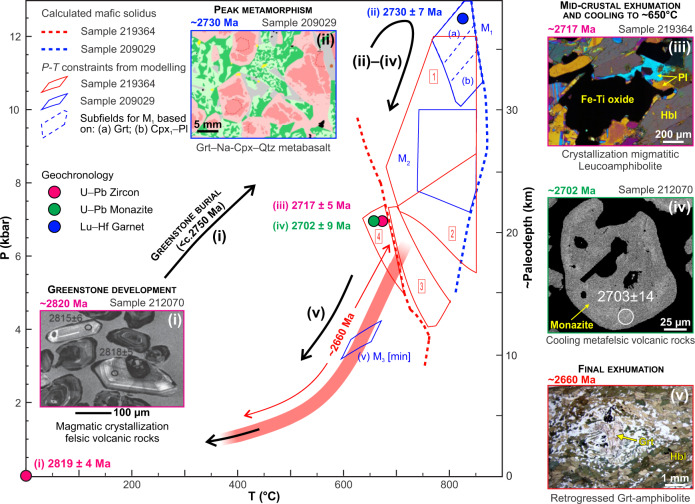
Metamorphic evolution of the Waroonga greenstone belt (WGB). Summary of the metamorphic evolution for the WGB, based on phase equilibrium modelling of samples 209029 and 219364. The five insets show the representative microstructures for each of the steps that we used to trace the *P–T–t* path. (i) Dating of euhedral zircons in sample 212070 define the formation age of the WGB; (ii) the peak assemblage in amphibolite clinopyroxene–garnet–quartz is well preserved in sample 209029, and dated by garnet-whole rock Lu–Hf isotopes in chemically similar sample 214208; zircon dating in leucoamphibolite (iii) and monazite dating in sample 212070 (iv) represent mid-crustal cooling of the WGB, during stepwise exhumation; (v) at c. 2660 Ma, exhumation was associated with the widespread development (in garnet amphibolite) of synkinematic hornblende, replacing both garnet (as shown in the micrograph) and clinopyroxene.

We produced *P–T* data from the dated migmatitic leucoamphibolite (sample 219364; Supplementary section 3). These results are integrated with existing metamorphic data from the WGB (sample 209029^35^, UA) to define a near-complete *P–T–t* path (Fig. 7).

Sample 209029 contains an interpreted peak mineral assemblage of clinopyroxene–hornblende–garnet–rutile–quartz–plagioclase. Phase equilibrium modelling of this assemblage^35^, based on the major oxide composition of this sample, combined with isopleths of garnet core compositions, constrains peak conditions to 11.2–13.0 kbar and 765–850 °C (M1a, Fig. 7). Slightly lower pressures of 10.0–11.5 kbar at similar temperatures^35^ are constrained by the integrated composition of former peak clinopyroxene (Cpx1) and plagioclase cores (M1b, Fig. 7). Leucoamphibolite sample 219364 contains the peak assemblage garnet–quartz–plagioclase–ilmenite–melt. Phase equilibrium modelling provides less well-constrained conditions that overlap with those from sample 209029 (Fig. 7). Predicted garnet compositions within this broad field do not vary and correspond well with measured values (Supplementary Fig. 12; Table 12).

The retrograde evolution of the rocks includes the pseudomorphic replacement of peak clinopyroxene (Cpx1) by retrograde clinopyroxene (Cpx2) with plagioclase and quartz (sample 209029, Fig. 4b, c), which is stable above 7 kbar and 740 °C (M2, Fig. 7). The growth of magnetite followed by hornblende (sample 219364) indicates a similar post-peak history of decompression, cooling and finally melt crystallization at 640–710 °C and 5.0–7.5 kbar (Supplementary section 3).

The youngest event recorded by the WGB corresponds to the synkinematic M3 assemblage defined by the pervasive development of hornblende ((v), Fig. 7). Syn- M3, synkinematic migmatitic amphibolite indicates that the first appearance of this assemblage must have occurred above the solidus (>650 °C) but at pressures below the M2 constraints of 7 kbar^35^ (Fig. 7).

## Discussion

**Fig. 8:**
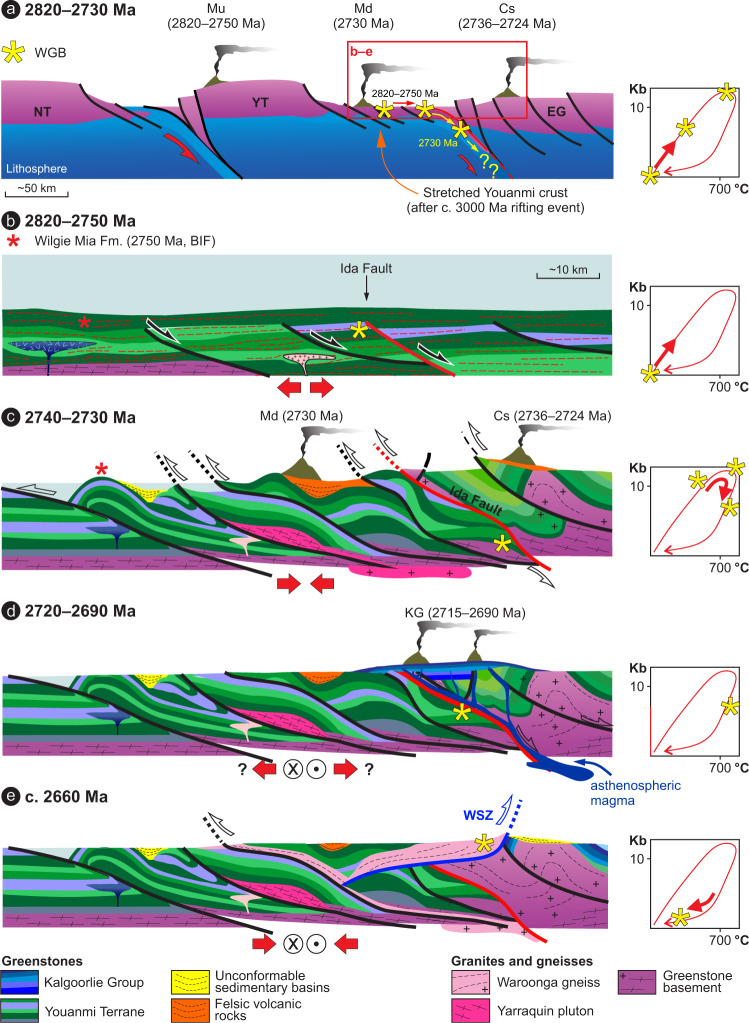
Tectonomagmatic evolution of the Waroonga greenstone belt (WGB) and the Yilgarn lithosphere. Cartoon outlining the tectonomagmatic evolution of the WGB and the Yilgarn lithosphere in the Meso- to Neoarchean. Note the change of scale from (**a**) to (**b**). **a** Long-term plate configuration characterized by the convergence between the Narryer (NT) and the Youanmi-Eastern goldfields terranes (YT and EG, respectively). Volcanism with an arc signature occurred in the Murchison^41^ (Mu), Marda^63^ (Md) and Cosmos^61^(Cs) areas. **b** The pre-orogenic period (until c.2750 Ma^40^) reflects the accumulation of a thick greenstone pile dominated by mafic–ultramafic volcanic rocks interlayered with BIFs, devoid of any clastic input^40^. The WGB developed at c. 2820 Ma, in the vicinity of the proto-Ida Fault. **c** Synmagmatic shearing along large-scale, east-dipping contractional structures, together with subaerial volcanism and development of high-energy sedimentary basins above a regional unconformity, mark the onset of the Neoarchean orogeny. Meanwhile, the WGB was buried to lower-crustal depths (12–13 Kbar^35^), along the Ida Fault, and subsequently partly exhumed to mid-crustal levels (7 kbar). Coeval volcanism with arc affinity occurred in the Cosmos area (Cs)^61^.**d** Mafic–ultramafic magmatism along the eastern margin of the Youanmi Terrane produced the Kalgoorlie Group greenstone sequence^10^ (KG). Asthenospheric magma (pictured in dark blue) was likely channelled along the main crustal-scale structure in the area, the Ida Fault. **e** Late-orogenic exhumation of the WGB took place along the Waroonga shear zone^35^, which channelled the emplacement of the syntectonic Waroonga Gneiss, transporting slivers of the WGB to their present position.

Our data, together with existing constraints, elucidate the Meso- to Neoarchean tectonic evolution of the WGB. Apart from having wide implications for the evolution of the Yilgarn Craton, this offers new insights into Neoarchean tectonics in general (Fig. 8). The WGB shares age, lithological and geochemical characteristics that are strikingly similar to greenstones from elsewhere in the Youanmi Terrane^10,39,41^ (Supplementary Figs 1 and 2). However, in a landscape dominated by low-grade metamorphic assemblages^10,39^, the high-grade metamorphic cycle (Fig. 7), together with the peculiar REE and O isotopic signatures in the amphibolite and BIF studied here, are so far unique to the WGB.

Strong enrichments in bulk rock REE, without coupled increases in Th and high field strength elements (HFSE), are not recognized primary features of basalt petrogenesis, and have not been recorded in unaltered Archean basalt^10^. Such compositional patterns, including the development of negative Ce anomalies, reflect post-magmatic net gains in REE, with only slight to moderate fractionation of LREE/HREE ratios. Similar enrichments in basalt may result from near-surface hydrothermal/metasomatic fluid interactions, fixing REE in secondary phosphates^51,52^, particularly within major shear zones^53^. The observed similarities between UA and EA whole-rock major element, Nb and Th contents, but striking whole-rock REE variations that are matched by respective garnet and amphibole compositions, further suggest that whole-rock REE enrichment in EA predated peak metamorphism and garnet growth. The absence of isotopic zoning (Fig. 5c) suggests that garnet grew in an environment that attained its oxygen isotope composition before garnet growth. Notably, garnet typically attains lower δ^18^O isotope signatures compared to the whole-rock values, due to isotope fractionation between other minerals, which is controlled by oxygen bond types^54^. Modelling has shown that the maximum oxygen isotope fractionation between whole rock and garnet at high temperature (>400 °C) is <2‰, with particularly low fractionation in mafic rocks that are poor in quartz and calcite^44,55^, such as the amphibolites studied here. The whole-rock δ^18^O value of EA sample 155899, at the time of garnet formation, therefore likely overlapped with a mantle-like δ^18^O signature (Fig. 5c). In contrast, the low δ^18^O values garnet in the unenriched amphibolite 214208 (~1.3‰, Fig. 5c), imply a distinctly sub-mantle whole-rock δ^18^O value of <3.3‰; at the time of garnet formation. Such an ^18^O-depleted composition is consistent with intense, near-surface high-temperature (hydrothermal) interaction with an isotopically light fluid, such as seawater or meteoric fluids^45^. We suggest that this garnet δ^18^O preserves the seafloor alteration signal in the unenriched amphibolites, whereas the slightly heavier O isotope composition of the enriched amphibolites reflects later, lower temperature fluid-rock interactions that also concentrated the REE in these rocks.

Since most greenstone sequences worldwide endured prolonged residence at (or near to) the ocean floor^10^, this history alone cannot explain the peculiar geochemical and isotopic signatures we document here. The requirement for locally intense hydrothermal alteration, combined with the observation that this alteration, characterized by addition of REE but not HFSE, occurs along major shear zones^53^, points to alteration of the WGB along a major structural discontinuity that may have corresponded to the Ida Fault (Fig. 8b), whose early activity dates back to c.3050 Ma^38^. Therefore, we suggest that the burial of the WGB started along a pre-existing weakness, an important prerequisite for any type of subduction initiation^56^.

Stratigraphic, structural and geochronological data indicate that the development of contractional structures in the Yilgarn Craton postdated the c. 2766–2747 Ma episodes of regional-scale granitic diapirism^20^. Furthermore, the regionally extensive, deep-marine BIFs of the c. 2750 Ma Wilgie Mia Formation, which were deposited during prevailing extension^39,40^, were then uplifted and eroded in the c.2734–2725 Ma interval during which a regional-scale unconformity developed^40^. Combined stratigraphic and structural data also indicate that the main regional structures in the central portion of the Yilgarn Craton studied here formed at c. 2730 Ma^57^, as part of the prominent Yilgarn-wide episode of crustal thickening. Thickening was accommodated within crustal-scale shear zones at the c. 2730 Ma onset of the Yilgarn orogeny^40^ (Fig. 8c), and ascribed to the collision between the Narryer and Youanmi terranes^38^ (Fig. 8a). Overall, these data indicate that burial of the WGB started after 2750 Ma (Fig. 8b), and that peak conditions of ~13 kbar were reached by 2730 ± 7 Ma, as revealed by our Lu–Hf garnet dating (Figs. 6d and 8c). Thus, there is a good correlation between the timing of collision between the Narryer and Youanmi blocks, craton-wide crustal thickening and high-P metamorphism in the WGB (Fig. 8a and c). We interpret the regionally preserved, gently east-dipping shear fabric that is exposed in low-grade greenstones along the footwall of the Ida Fault (Figs. 1c, d, and 2), and which characterizes the lithosphere-scale architecture of the whole craton (Fig. 1b), to represent the expression of such a convergent tectonic setting (Fig. 8c).

Exhumation to mid-crustal levels (~7 kbar, as recorded by the leucoamphibolite sample 219364, Figs. 6c and 7) was achieved by 2717 ± 5 Ma, essentially during the same contractional event that marked the onset of the Yilgarn orogeny^40^. This zircon age of the migmatitic fabric recorded within the leucoamphibolite sample (Fig. 6c) coincides with the onset of eruption of the mafic–ultramafic Kalgoorlie Group (c. 2714 Ma^58^). Therefore, we interpret this migmatitic event as the signature of the crustal-scale thermal antiform associated with lithospheric thinning and the transfer of voluminous, asthenosphere-derived mafic–ultramafic magma^10^ (Fig. 8d). The monazite U–Pb age (c. 2700 Ma, sample 212070, Fig. 6b) likely marks the same thermal event, at a similar mid-crustal level (Fig. 7). About 50–60 Myr later, c. 2660 Ma exhumation along the WSZ (Fig. 8e) to the near surface commenced at the same mid-crustal level (~7 kbar, Fig. 7), and was achieved during a major synmagmatic episode of transpressional tectonics^35^.

**Fig. 9:**
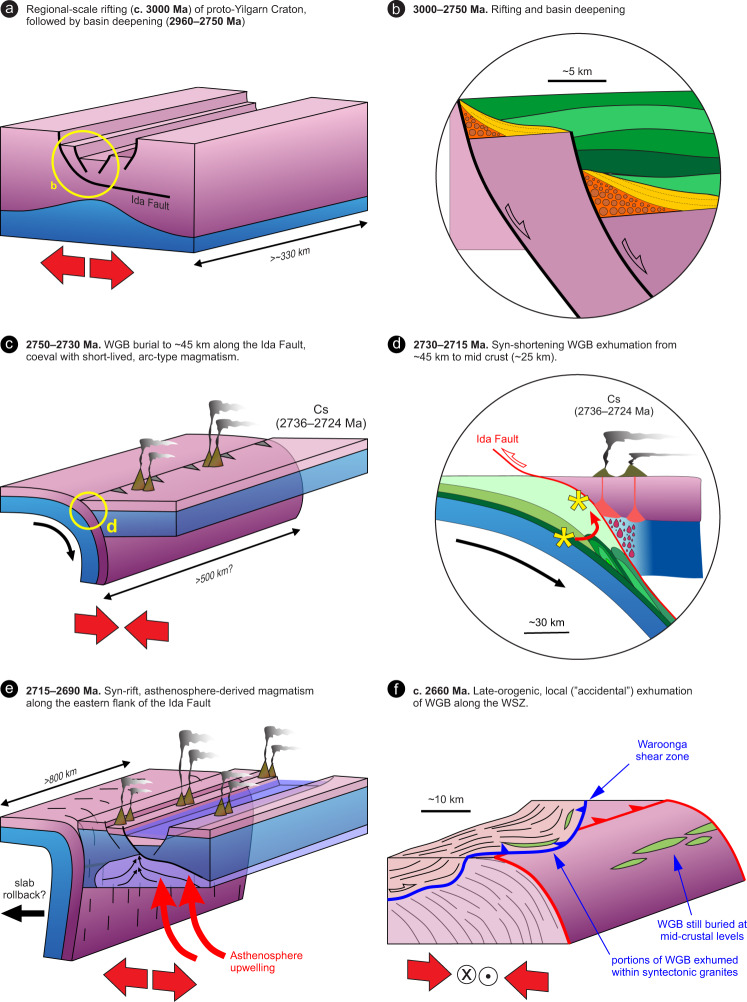
Tectonic evolution of the central portion of the Yilgarn Craton. Meso- to Neoarchean tectonic evolution of the central portion of the Yilgarn Craton, with emphasis on the along-strike structural continuity. **a** C. 3000 Ma, regional-scale rifting occurred in the central part of the Yilgarn Craton. The minimum size of the rift-related structures is inferred by the current along-strike exposure (~330 km) of the syn-rift quartzite (Fig. 1a). **b** Detail from **a** showing the syn-rift deposition of conglomerate (orange) and quartzite (yellow) at c. 3000 Ma, followed by the 2960–2750 Ma post-rift, deep-marine sequence (green), of prevailing basalt and Banded Iron Formation (BIF). **c** 2750–2730 Ma burial of the eastern margin of the Youanmi Terrane under the Eastern Goldfields Superterrane (EGST). The current extension of the Ida Fault (~500 km) suggests that the buried margin extended for several hundred km along strike. **d** Detail from (**c**) showing the post-peak, syn-shortening exhumation of the Waroonga greenstone belt (WGB) (asterisk) to mid-crustal levels, broadly coeval with the Arc-type magmatism in the hanging wall. **e** 2715–2690 Ma asthenospheric magmatism occurred along a rift zone that was ~800 km along strike, as suggested by the current distribution of the Kalgoorlie Group, in the hanging wall of the Ida Fault. Asthenosphere upwelling may have been triggered by slab rollback or break off. **f** Exhumation of portions of the WGB took place along the Waroonga Shear Zone (WSZ), a structure that is unrelated to the Ida Fault. Since this structure provided ~10 km uplift at c. 2660 Ma, other portions of the WGB are inferred to now lie at mid-crustal levels.

In the central part of the Yilgarn Craton, the c. 3000 Ma syn-rift quartzites have a current along-strike exposure of ~330 km, and the Ida Fault has a current along-strike continuity of ~500 km (Fig. 1). This suggests that both the Mesoarchean rift-related structures (Fig. 9a and b), and the Neoarchean convergent margin (along which the WGB was buried to lower-crustal conditions), were craton-scale features, several hundreds of km in size (Fig. 9a–d). Furthermore, given that the 2715–2690 Ma Kalgoorlie Group has a present-day along-strike extension of ~ 800 km, the rift-related structures that controlled the development of this asthenosphere-derived volcanic succession^10^ were likely of comparable size (Fig. 9e). Overall, the deformation style pictured here, which includes regional-scale shearing along crustal-scale shear zones (Figs. 1, 8 and 9), is indicative of relatively high integrated lithospheric strength^15,35,59^, suggesting that burial of the WGB occurred through some form of subduction, rather than through gravity-driven drip tectonics. The latter typically produced dome-and-keel map patterns with minimal along-strike continuity^60^, in weak lithospheric domains^19,59^, which are incompatible with the overall lithospheric geometries and structural style that typified the Yilgarn Craton since at least c. 3000 Ma (Fig. 9). The occurrence of 2736–2724 Ma volcanic arc-like successions in the hanging wall of the Ida Fault^61^ (Fig. 8c) suggest that the burial of the WGB took place in the context of an east-dipping subduction setting, consistent with the coeval subduction zone inferred to have existed at that time along the western margin of the craton^26,38,41^ (Fig. 8a), and with the east-dipping fabric preserved throughout the craton (Fig. 1b).

Assuming that the ~13 kbar peak pressure recorded by the WGB corresponds to burial to ~45 km (considering an average crustal density of 2.8 gr/cm^3^), and that burial occurred within a maximum time span of 20 Myr (from ≤ 2750 to 2730 Ma), minimum burial rates in the order of ~2.3 mm/yr can be calculated. This value is about one order of magnitude lower than Phanerozoic rates of subduction^62^, although burial of the WGB may have started well after c. 2750 Ma, such that burial rates were higher. Peak conditions reflect an apparent thermal gradient of 70 °C/kbar, which is in line with intermediate T/P style of metamorphism that is widely registered in the Neoarchean rock record^5^ (Fig. S14). The subsequent exhumation to ~25 km, which was achieved by 2715 Ma (Fig. 7), was essentially coeval with 2736–2724 Ma arc-like volcanism that took place during active convergence (Fig. 9d), with minimum uplift rates in the order of 1.3 mm/yr. Notably, exhumation from peak conditions took place along a *P–T* path approaching isothermal decompression (Fig. 7), consistently with fast, tectonic exhumation via return flow, within an active convergent margin^59^.

From a craton-wide perspective, by combining our data with those available from the literature, we identify two broadly coeval, east-dipping subduction zones that offer a strikingly contrasting geological record. In the west, along the Narryer–Youanmi boundary (Fig. 8a), 2820–2730 Ma protracted subduction^26^ generated one of the most complete Archean arc-related sequences, with compositional ranges approaching those of modern-style subduction settings^10,41,63^, but with no record of exhumed high-pressure rocks. Conversely, burial along the Ida Fault has left a short-lived supracrustal arc signature (2736–2724 Ma)^61^ (Fig. 8c), but has exposed greenstones previously buried to lower-crustal depths. Assuming that such contrasting records do not reflect preservation bias, they may represent one of the hallmarks of the transitional tectonic style that typified the Neoarchean Earth^2,12^. Overall, burial along late-Archean subduction zones bore similarities with modern subduction: the transformation of basalt to dense garnet-bearing rocks including eclogite would have been the primary driver for Archean subduction, as it is today. However, the burial of hotter (weaker) mafic crust implied that the early stages of subduction would have been inherently more unstable than today, favoring episodes of slab breakoff and slab rollback^11^. These would have in turn triggered vigorous asthenosphere upwelling, driving deeply sourced basaltic magmatism, and suppression of arc magmatism^64^, which accounts for the two types of volcanism recorded in the hanging wall of the Ida Fault (Fig. 9d and e), and in many greenstone sequences worldwide^10,11^. Overall, short-lived subduction was likely the prevailing modus operandi during the late Archean^9,11^, consistent with the prevalence of subduction initiation sequences in the Archean crustal record^10^.

Our data also show that post-peak exhumation to mid-crustal depths along the WSZ was followed by a 50–60 Myr-long period of apparent tectonic quiescence, before final exhumation to depths of a few km (Figs. 8e and 9f), which provided ≥10 km of uplift at c. 2660 Ma^35^. Remarkably, greenstones slices within the WGB are only exposed along a ~20 km-long (along-strike) segment of the WSZ (Fig. 3a). We infer these slices to represent a minor component of the greenstones originally buried along the convergent margin, which are inferred to now lie within the mid-crustal portions of the Ida Fault (Fig. 9f). Furthermore, between the exposed portions of the WGB, relatively pristine peak assemblages are only preserved locally, within a km-scale segment of the shear zone (Fig. 3c); elsewhere along the WSZ, amphibolite is typically strongly retrogressed and amphibole rich, preserving only rare garnet fragments and/or garnet pseudomorphs^35^ ((v) in Fig. 7). Although the presence of a hot and magma-rich medium facilitated greenstone exhumation along the WSZ, it also favoured pervasive retrogression of the high-pressure, anhydrous assemblages.

The timing, rate and *P–T* path of the first stage of WGB exhumation (from lower to mid-crustal levels) are all comparable to those typically observed in modern subduction zones^65^. This suggests that exhumation processes that operated at late Archean convergent margins may have been similar—at least locally—to those that characterize Phanerozoic subduction zones. However, while exhumation of high-P rocks along modern convergent margins is typically completed during active subduction^65^, in the studied rocks final exhumation (from the mid crust to near-surface levels) was achieved through melt-assisted transpression^35^, during a tectonic episode that occurred c. 60 Myr after that responsible for burial and early exhumation (Figs. 8e and 9f). A similar—and coeval—tectonic evolution is recorded in West Greenland (North Atlantic Craton), where c. 2715 Ma exhumation of greenstone to mid-crustal levels (~ 5 kbar) took place shortly after peak metamorphism (at ~10 kbar), and was followed by slow isobaric cooling^66^.

Both the tectonic style and the time scale of the burial–exhumation cycle described here differ substantially from those typifying sagduction-driven burial–exhumation cycles associated with gneiss domes, which may occur in a few Myr only^19^. This should not be surprising since, as outlined earlier, the transpressional orogen exposed in the Yilgarn Craton and the archetypal dome-and-keel architecture of the Pilbara Craton lie at the opposite ends of the spectrum, in terms of Archean tectonic styles.

The oldest-known, laterally continuous belt of exhumed high-pressure rocks corresponds to the Paleoproterozoic (c. 2000 Ma) Usagaran belt of Tanzania^67^. Before that, short-lived, unstable subduction was capable of producing eclogites along Archean convergent margins^34^, but exhumation processes in those environments were likely less efficient than today, so that complete exhumation of high-pressure rocks was much rarer. Furthermore, those rare rocks had lower preservation potential than today, if exhumation was assisted by a low-viscosity, buoyant, hot and fluid-rich medium such as partially molten rocks, which would favour retrogression of anhydrous assemblages.

Fast (tectonic) exhumation requires efficient strain localization along large-scale shear zones^15^, which in turn needs to be supported by a stiff lithospheric mantle^59^. The mixed signals that we detect in the late Archean rock record reflect a transitional tectonic style, consistently with the progressive cooling of the Earth’s mantle^11^.

Although our study cannot directly prove (or disprove) the existence of plate tectonics in the late Archean, our results are consistent with those emerging from other Neoarchean environments. Late Archean geological processes that include subduction and high-grade metamorphism during terrane assembly along convergent margins have been inferred in the Superior Province^25^, and in the North Atlantic^34,66^ and Dharwar^68^ cratons. The resulting picture describes the Neoarchean as a transitional and highly dynamic phase of Earth’s evolution, which has left a mixed signal in the geological record (reflecting variably short-lived subduction events^11^, and variably efficient exhumation styles) and led to the progressive establishment of a global network of plates and long-lived subduction zones—the critical ingredients of modern-style plate tectonics^9^—during the early Paleoproterozoic^12,69^.

## Methods

### Whole-rock major and trace element analyses

Whole-rock major and trace elements were determined either at Bureau Veritas, Perth, Western Australia. Major and minor elements (Si, Ti, Al, Cr, Fe, Mn, Mg, Ca, Sr, Ba, Na, K and P) were determined by X-ray fluorescence (XRF) spectrometry on a fused glass disk and loss on ignition (LOI) was determined by thermogravimetric analysis. The concentrations of Ag, As, Ba, Be, Bi, Cd, Ce, Co, Cr, Cs, Cu, Dy, Er, Eu, Ga, Gd, Ge, Hf, Ho, La, Lu, Nb, Nd, Ni, Pb, Pr, Rb, Sc, Sm, Sn, Sr, Ta, Tb, Th, Tl, Tm, U, V, W, Y, Yb, Zn and Zr were all determined by laser ablation inductively coupled plasma mass spectrometry (LA-ICP-MS) on a fragment of the same glass disk used earlier for XRF analysis. Data quality was monitored by blind insertion of sample duplicates, internal reference materials, and the certified reference material OREAS 24b. Bureau Veritas also included duplicate samples, certified reference materials (including OREAS 24b), and blanks. Total uncertainties for major elements are ≤1.5%, those for minor elements are <2.5% (at concentrations >0.1 wt.%) and those for most trace elements are ≤10% (Lu ± 20%).

### Major element analyses and quantitative element mapping via electron probe microanalyzer (EPMA)

Quantitative element analyses on thin section (Supplementary Table 2) were acquired on a JEOL JXA8530F electron probe micro-analyzer instrument, equipped with five tunable wavelength-dispersive spectrometers, at the Centre for Microscopy, Characterisation and Analysis (CMCA) at The University of Western Australia. Operating conditions were 40° take-off angle, and 15 keV of beam energy. The beam current was 20 nA, and the beam diameter was 5 µm. Elements were acquired using analyzing crystals LiF for Fe kα, Mn kα, PETJ for Ca kα, K kα, Ti kα, Cr kα, P kα, and TAP for Na kα, Si kα, Mg kα, Al kα. The standards used for instrument calibration were periclase for Mg, San Carlos olivine for Si, Durango apatite for Ca and P, corundum for Al, jadeite for Na, magnetite for Fe, Mn metal for Mn, Cr_2_O_3_ for Cr rutile for Ti, and orthoclase for K. The on-peak counting time was 20 s for all elements and Mean Atomic Number (MAN) background corrections used throughout^70^. The sample and standard intensities were corrected for deadtime. The intensity data were corrected for Time Dependent Intensity (TDI) loss (or gain) using a self-calibrated correction for Na kα, Si kα, Fe kα, K kα. The matrix correction method was ZAF^71^ and data reduction used the Probe for EPMA software package. Typical detection limits ranged from 0.01 weight percent for Al kα to 0.02 for Na. Oxygen was calculated by cation stoichiometry and included in the matrix correction.

Quantitative map acquisition was performed using the Probe Image® software for X-ray intensity acquisition. The beam current was 40 nA with a 40 msec per pixel dwell time and a 2 × 2 µm pixel dimension. Image processing and quantification was performed off-line with the CalcImage® software, the calibration procedure outlined above and output to Surfer®. Supplementary Fig. 8 shows quantitative EPMA maps of individual garnets, while Supplementary Fig. 9 shows point analyses along transects in individual garnet crystals.

### Trace-element analyses via LA-ICP-MS

Garnet from sample 214208 were analysed on polished thin sections at the University Western Australia, Perth (Australia), following the same profiles that were analysed by EPMA for their major element concentrations. LA-ICP-MS analyses followed the procedure described in^72^ and were conducted using an X-series II quadrupole ICP-MS attached to an Analyte G2 Excimer laser system (193 nm wavelength) with a standard dual volume cell. The He carrier gas was set at 1.0 l/min (MFC1 = 0.6 l/min and MFC2 = 0.4 l/min) and was mixed with a nebulizer flow of argon (0.7 l/min) in a glass mixing bulb. N_2_ was added with a steady flow of ~5 ml/min in order to enhance sensitivity and reduce oxide production before being introduced into the plasma. The mass spectrometer was tuned to maximum sensitivity at plasma conditions of Th/U ~1 in a NIST610 glass, and the production of molecular oxide species was monitored by maintaining a low ThO^+^/Th^+^ ratio <0.25%. The laser ablation repetition rate was 10 Hz, with a laser fluence of 5 J/cm^2^. A circular spot of 50 μm diameters was used for all unknowns and standards. The dwell time for each element was set at 10 ms. A 30 s background was collected prior to each analysis. Element concentrations were reduced with the Iolite software package^73^ using bracketing analysis of NIST610 as an external standard with the reference concentrations taken from^74^. CaO determined by EPMA was used as the internal standard values for each analysed grain and NIST612 was analysed and used as a secondary standard. Supplementary Table 3 shows all LA-ICP-MS data, including standard reference material, while Supplementary Fig. 10 and 11 show analytical profiles of individual garnet grains.

### Oxygen isotope analyses via SIMS

In-situ ^18^O/^16^O measurements in garnet were carried out using a Cameca IMS 1280 housed at the Centre for Microscopy Characterisation and Analysis at the University of Western Australia. O isotope measurements were performed on thin sections cut with a precision saw and mounted in epoxy, together with pre-polished blocks containing a range of reference materials for O isotope measurements in garnet (as detailed below).

The sample mounts were thoroughly cleaned with detergent, ethanol and distilled water in an ultrasonic bath and coated with a 30nm-thick Au coating prior to SIMS analyses. During the analyses, the sample surface was sputtered over a 10 × 10 µm area with a 10 kV, gaussian Cs^+^ beam with intensity of 2.5 nA and a total impact energy of 20 keV. An electron gun was used to ensure charge compensation during the analyses. Secondary ions were admitted within a 110 µm entrance slit and focused in the centre of a 4000 µm field aperture (x 130 magnification). Energy filtering was applied using a 30 eV band pass with a 5 eV gap toward the high-energy side. ^16^O and ^18^O were collected simultaneously in Faraday cup detectors fitted with 10^10^ Ω (L’2) and 10^11^ Ω (H1) resistors, respectively, and operating at a mass resolution of ~2430. The magnetic field was regulated using NMR control.

Each analysis includes a pre-sputtering over a 15 × 15 µm area during 40 s, followed by the automatic centreing of the secondary ions in the field aperture, contrast aperture and entrance slit. Each analysis then consists of 20 four-second cycles, which give an average internal precision of ~0.16‰; (2 SE). The analytical session was monitored in term of stability using at least two bracketing standards (UWG-2) every 5 to 6 sample analyses. The spot-to-spot reproducibility on UWG-2 was ~0.4‰; (2 SD) during the analytical session. For garnet, the correction for instrumental mass fractionation has to account for the bias, or matrix effect, related to the difference in chemistry between the unknowns and the reference materials^75–77^. In absence of uvarovite and andradite components in garnet (as it was confirmed by EPMA data in the present study, Supplementary Table 2), the grossular component (*X*_Gr_) creates the largest bias on oxygen isotope measurements by SIMS^75–77^. Therefore, six garnet reference materials, with different *X*_Gr_, were analysed at the beginning, middle and end of the analytical session, for each mount, to model the matrix effect and correct the measurements of the unknowns. Oxygen isotope analyses in the samples were acquired next to the spot locations where EPMA measurements were performed for the matrix correction (Supplementary Figs 6 and 7). The garnet reference materials used in this study were the same for the 2 mounts: 10691, 2B3, GRS-SE and UWG2^75,76^ and ALM-GEM (in house, *X*_Gr_ = 0.01, d^18^O = 6.79‰;). For both mounts, the relationship between bias and *X*_Gr_ can be fit with a parabola, with a correlation coefficient (*r*^2^) better than 0.97, returning a regression residual of ~0.3 per mil, in the range of the external reproducibility observed on UWG-2.

The uncertainty on single spot analyses reflects the residual observed on the calibration curve, which represent the average difference between the model and the measurements for the matrix effect calibration curve and the external reproducibility on UWG-2. Supplementary Table 4 presents the raw ^18^O/^16^O ratios and the corrected δ^18^O values (quoted with respect to Vienna Standard Mean Ocean Water or VSMOW in per mil).

### Rare earth element analyses

REE analyses of garnet and hornblende were performed at the John de Laeter LA–ICP–MS facility using a Resonetics M-50 193 nm excimer laser with an Agilent 7700 mass spectrometer. Both minerals were analysed in polished thin sections, using a beam diameter of 23 μm and a repetition rate of 5 Hz, which produced a laser power density of ~3 J/cm-2. Data was collected using time-resolved data acquisition and processed using the Iolite software package^78,79^. Total acquisition time per analysis was 80 s including 30 s of background time and 40 s of sample ablation, followed by a 10 s washout period. Calibration was performed against the NIST 610 standard glass using the coefficients of^80^. Stoichiometric Si was used as the internal standardization element for garnet (18%) and the Si content of hornblende as determined by EPMA. Precision based on repeated analysis of standards is 5–10%, with detection limits for REE in this study ranging from 0.1 to 0.5 ppm.

### Zircon and monazite geochronology

The zircons were separated after rock crushing using conventional heavy liquid and magnetic properties. The grains were mounted in epoxy resin and polish down to expose the near equatorial section. Cathodoluminescence (CL) investigation was carried out on a HITACHI S2250N scanning electron microscope supplied with an ellipsoidal mirror for CL at the Electron Microscopy Unit at the Australian National University in Canberra. Operating conditions for the SEM were 15 kV/60 µA and 20 mm working distance. Zircon crystals from sample 219364 were imaged for CL using a CamScan MV2300 at the Institute of Earth Sciences at University of Lausanne, Switzerland, working at 10 kV and a working distance of 10 mm. The zircon crystals were analysed for U-Th-Pb using the sensitive high resolution ion microprobes (SHRIMP II, sample 212070 and 212796) at the Australian National University, and the Cameca IMS 1280-HR at the University of Lausanne (sample 219364). Instrumental conditions and data acquisition were generally as described by^81^ for SHRIMP and by^82^ for Cameca IMS 1280. The data were collected in sets of six scans throughout the masses and a reference zircon was analysed each fourth analysis. U-Pb data were collected over two analytical sessions having calibration uncertainties of 1.0 and 2.1% (2 sigma, Pb/U versus UO/U calibration), which was propagated to single analyses. The measured ^206^Pb/^238^U ratio was corrected using reference zircon FC1 (1099 Ma, REF) during SHRIMP analyses and zircon 91500 (1065 Ma^83^) during Cameca-1280 analyses, while FC1 zircon was used as secondary standard (obtained Concordia age 1094 ± 11 Ma). Monazite crystals were analysed using the SHRIMP II ion microprobe following similar analytical conditions as for zircon. Data were collected over a single analytical session with a calibration uncertainty of 1%, using Thompson Mine Monazite as standard (1766 Ma^84^). The analyses for zircon and monazite were corrected for common Pb based on the measured ^204^Pb according to^81^. Data evaluation and age calculation were done using the software Squid 2 and Isoplot/Ex^85^. The common Pb composition was assumed to be that predicted by^86^ model. Average ages are quoted at 95% confidence level (c.l.).

### Garnet Sm-Nd and Lu-Hf geochronology

We selected the garnet amphibolite sample 214208 for garnet Sm-Nd and Lu-Hf geochronology, following the methods described by^87,88^. In the first instance, pieces of texturally homogeneous rock, free of visible weathering or alteration, were pulverised to gravel-sized particles. From this, ~100 g of clean rock chips were selected for whole rock geochemistry, where the chips were rinsed in de-ionised water. A rotating splitter was used to obtain a 20 g aliquot of chips, which was then ground to powder in an agate ball mill. A second 10 g aliquot was prepared from small rock chips that lacked visible garnet (‘matrix’) and this was powdered separately. The remaining crushed material was disc-milled to a < 500 µm fraction for mineral separation. A garnet concentrate was prepared by standard hydrodynamic and magnetic separation techniques, and from this three aliquots of garnet grains of ~200–250 mg were handpicked under a binocular microscope, emphasising fragments of highest optical clarity and lack of inclusions. These garnet separates were firstly leached in 1 M HCl in an ultrasonic bath, rinsed with MilliQ water, and dried.

The garnet fractions and two ~250 mg whole-rock powders were subsequently dissolved in concentrated HF-HNO_3_ mixtures (10:1). The whole rock powders were digested at high pressure in a steel-jacketed PTFE (Teflon) dissolution vessel (Parr type bomb) at 150 °C for 1 week, whereas the garnets were dissolved in sealed Savillex beakers on a 120 °C hotplate for 3 days, to minimise the dissolution of older un-equilibrated accessory phases, such as zircon. Solutions (minus any solid residue in the garnet fractions) were dried and converted to chloride form using firstly a mixture of H_3_BO_3_ and 6 M HCl and secondly by transferring all samples in 6 M HCl to steel jacket digestion vessels and heating overnight at 150 °C. This yielded clear solutions that were then transferred back to Savillex beakers, spiked with mixed ^176^Lu-^180^Hf and ^149^Sm-^150^Nd tracer solutions and allowed to equilibrate on a hotplate for 24 h.

Procedures for chemical separation of Sm, Nd, Lu, and Hf using cation exchange chromatography followed the methodology outlined by^89^. The first stage columns contained 10 ml of BioRad AG50w-X12 resin, onto which samples were loaded as 3 ml 1.0 M HCl/0.1 M HF solutions. The HFS elements were subsequently eluted with 6 ml of 1.0 M HCl/0.1 M HF. The sample matrix was washed from the column with 60 ml of 1.0 M 2.5 M HCl, before a HREE cut was collected using a further 80 ml of 2.5 M HCl. Upon passage of another 25 mL of 2.5 M HCl, the LREE, including Sm and Nd, were eluted in 50 mL of 6.0 M HCl. The HFSE and both the HREE and LREE cuts were then dried for subsequent purification.

The HFSE cut from the first column was dried, re-dissolved in 5 ml of 2.5 M HCl and loaded onto a 0.9 mL column packed with Eichrom Ln-spec resin. The resulting procedure followed the methods of^90^. The column was first washed with 10 ml of both 2.5 M HCl and 6.0 M HCl, after which Ti was removed from the other HFSE with a 0.45 M HNO_3_/0.09 M citric acid/1%H_2_O_2_ mixture. A 6 M HCl/0.06 M HF solution was then used to wash Zr from the column, before Hf was eluted using 5 mL of 6.0 M HCl/0.4 M HF. As a final purification step to remove any remaining REE, this Hf cut was dried, and then loaded as a 0.2 mL 1.0 M HCl/0.1 M HF solution onto a miniaturized (0.6 mL) version of the first stage column. The Hf was collected using 0.6 mL of 1.0 M HCl /0.1 M HF.

The LREE eluate from the first column stage was dissolved in 1 mL of 0.14 M HCl and loaded onto a column filled with 1.7 ml of LnSpec resin for separation of Sm and Nd. Initially, 45 ml of 0.14 M HCl was passed though the column, before Nd was eluted in 40 ml 0.14 M HCl. Sm was subsequently eluted using 10 ml of 0.4 M HCl. The HREE fraction from the first stage column was dissolved in 0.5 mL of 2.5 M HCl and loaded onto 0.9 mL columns containing Ln-spec resin. The resin was then rinsed with 35 mL of 2.5 M HCl to remove the majority of the Yb, before Lu and the remaining Yb was eluted using 5 mL of 6.0 M HCl.

The purified Sm, Nd, Hf and Lu cuts were dried and dissolved in 2% HNO3 in preparation for mass spectrometry. All isotopic analyses were performed on the ThermoFinnigan Neptune multicollector ICP-MS at Washington State University, following procedures outlined by^91^ and by^92^. Nd analyses were corrected for mass fractionation using ^146^Nd/^144^Nd = 0.7219 and normalized using the JNdi reference solution. Sm analyses were corrected for fractionation using ^147^Sm/^152^Sm = 0.56081. Whole-rock Hf analyses were corrected for mass fractionation using ^179^Hf/^177^Hf = 0.7325^93^ and normalized using the JMC-475 reference Hf solution. Lu measurements were made according to the procedure of^91^. Mass fractionation corrections were made using the exponential law. Over the course of the study, the mean measured ^143^Nd/^144^Nd value for the JNdi solution was 0.512088 ± 0.000016 (2σ, *n* = 10), and the mean ^176^Hf/^177^Hf value for the JMC 475 Hf standard was 0.282141 ± 0.000008 (2σ, *n* = 9).

The Lu-Hf and Sm-Nd isochron dates were calculated with a ^176^Lu decay constant of 1.867 × 10-11 a^−1,^^94^ and ^147^Sm decay constant of 6.54 × 10–12 a^−1^ ref. 95 Isochron regressions were performed with Isoplot/Ex^85^.

### Metamorphism

Mineral chemistry data from sample 219364 was acquired using a Cameca SX-Five electron probe microanalyzer (EPMA) at Adelaide Microscopy, University of Adelaide, equipped with 5 tuneable wavelength-dispersive spectrometers. Instrument operating conditions were 15 kV/20 nA with a defocused beam of 5 µm. Beam damage and alkali element migration in silicate analyses were minimized via use of a defocused electron beam. Calibration was performed on certified synthetic and natural mineral standards from Astimex Ltd and P&H Associates. Mean Atomic Number (MAN) background correction was used^96^. Data calibration and reduction was carried out in Probe for EPMA, distributed by Probe Software Inc.

The bulk rock composition used for phase equilibria modelling was determined by X-ray fluorescence spectroscopy at Genalysis Laboratory Services, Australia, together with loss on ignition (LOI). FeO content was analysed by Fe^2+^ titration also at ALS Chemex, Australia, and Fe_2_O_3_ calculated by difference.

## Supplementary information

Supplementary Information

Description of Additional Supplementary Files

Supplementary Data

## Data Availability

All the data that support the findings of this study have been deposited in the Open Science Framework, and are available at the following link: https://osf.io/ykrqf/?view_only=118264df69a142f19c9918ce243b1d28.
