# Supplementary material for: Greenstone burial–exhumation cycles at the late Archean transition to plate tectonics
Source: Nat Commun. 2022 Dec 22;13:7893. doi: 10.1038/s41467-022-35208-2 (PMC9780361; doi:10.1038/s41467-022-35208-2)
Supplement: Supplementary file 1 — Supplementary Information [file 41467_2022_35208_MOESM1_ESM.docx]

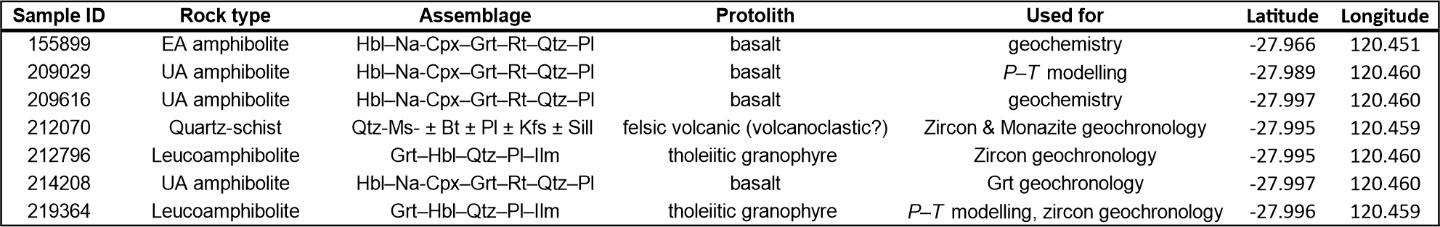


**Supplementary Table 1**. List of samples from the Waroonga Greenstone Belt used in this study. The additional amphibolite and banded iron formation (BIF) samples used in the geochemistry section are listed in Supplementary Dataset 2 and 6.

**Supplementary section 1. Geochemistry and O isotopes in garnet**

**
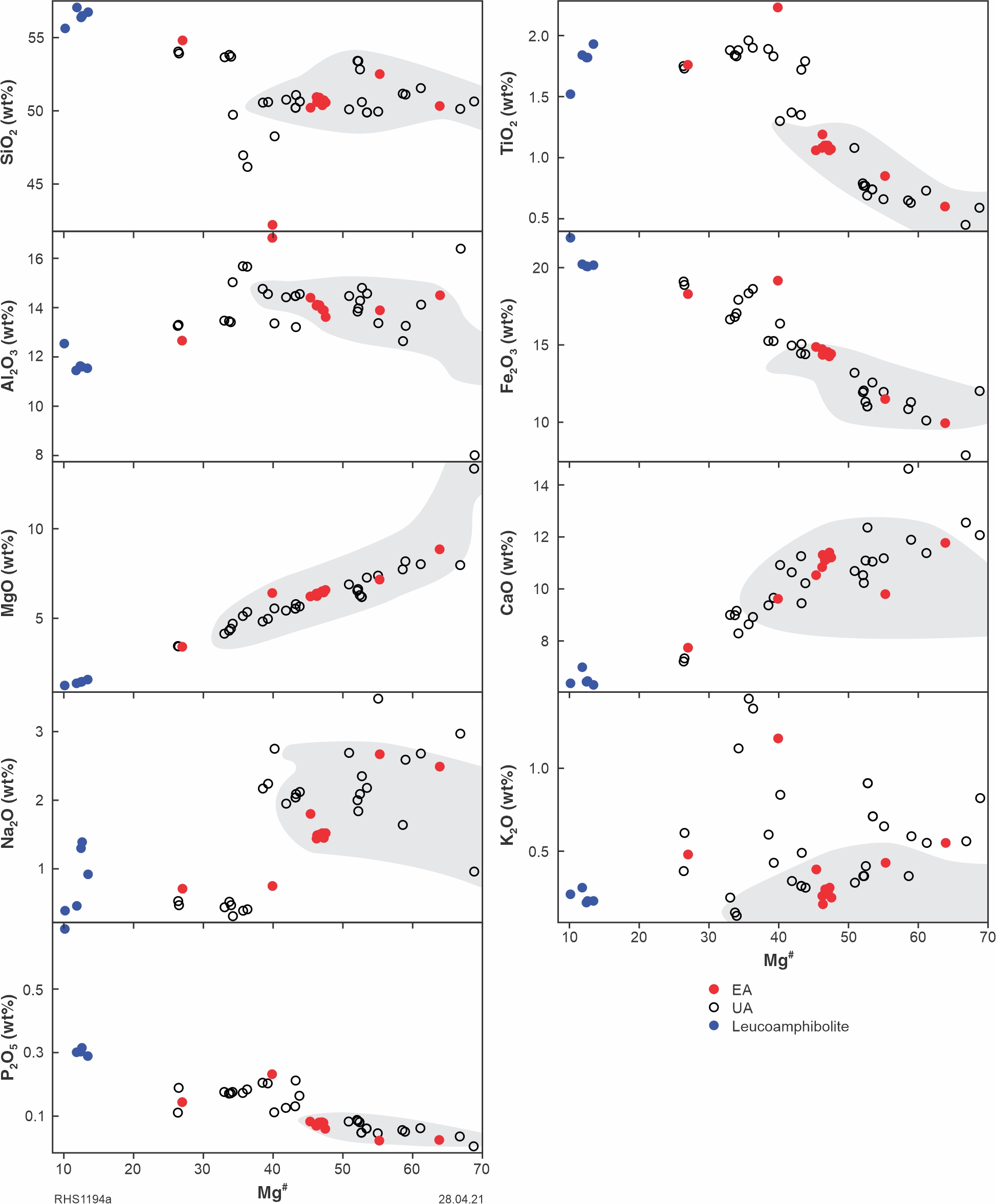
**

**Supplementary Figure 1.** **Compositional variation diagrams of major elements in amphibolite**. Compositional variation diagrams showing variation in major element concentrations with Mg# [= molecular ratio of MgO/(MgO+FeO) where FeO represents all Fe as Fe2+] for all amphibolite samples. The corresponding analyses are shown in Supplementary Datasets 2 and 3. Grey areas represent the compositional field for typical basaltic rocks of the Yilgarn Craton (Supplementary Dataset 3).


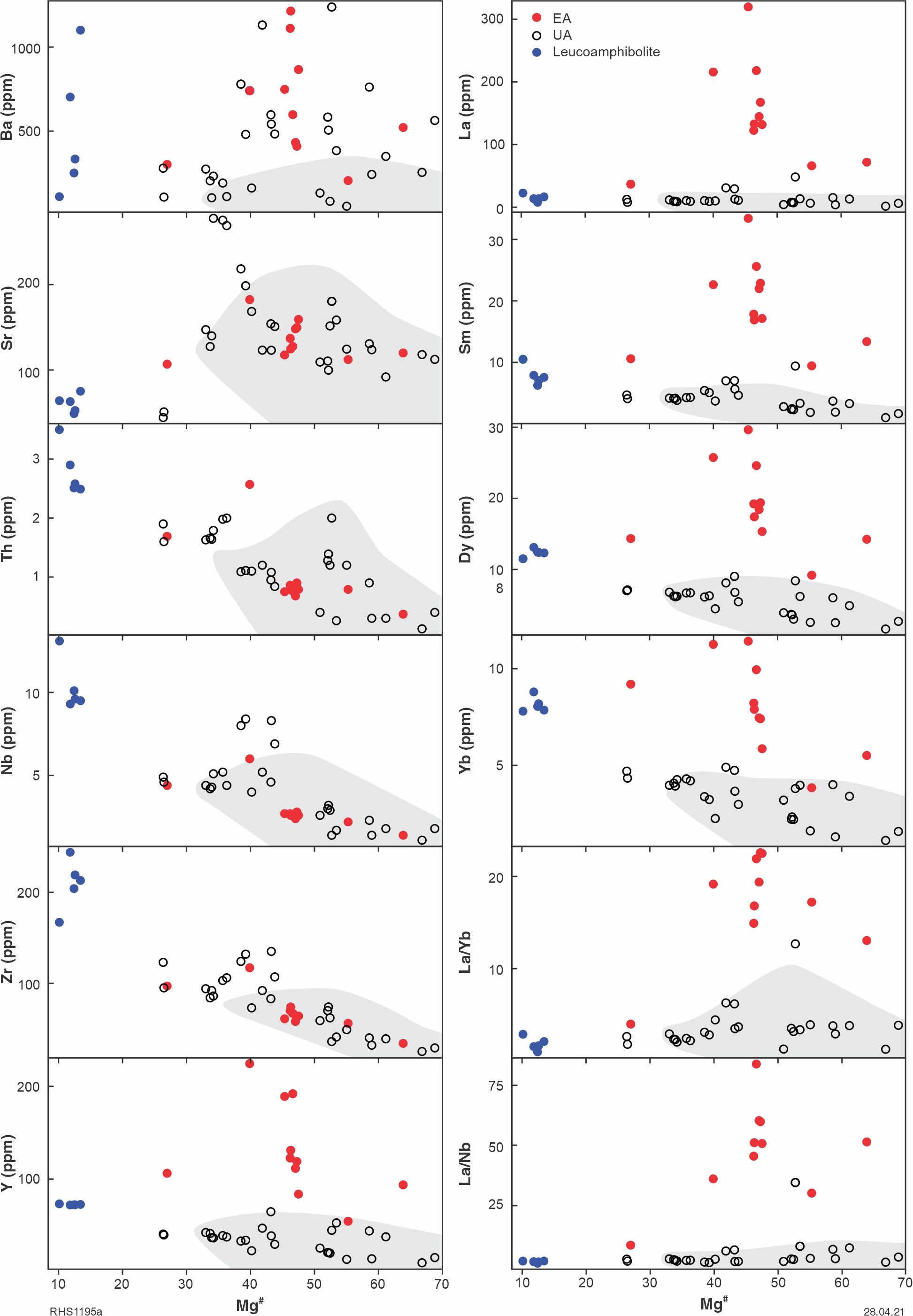


**Supplementary Figure 2.** **Compositional variation diagrams of trace elements in amphibolite**. Compositional variation diagrams showing variation in trace element concentrations and trace element ratio with Mg# [= molecular ratio of MgO/(MgO+FeO) where FeO represents all Fe as Fe2+] for all amphibolite samples. The corresponding analyses are shown in Supplementary Dataset 2 and 3. Grey areas represent the compositional field for typical basaltic rocks of the Yilgarn Craton (Supplementary Dataset 3).


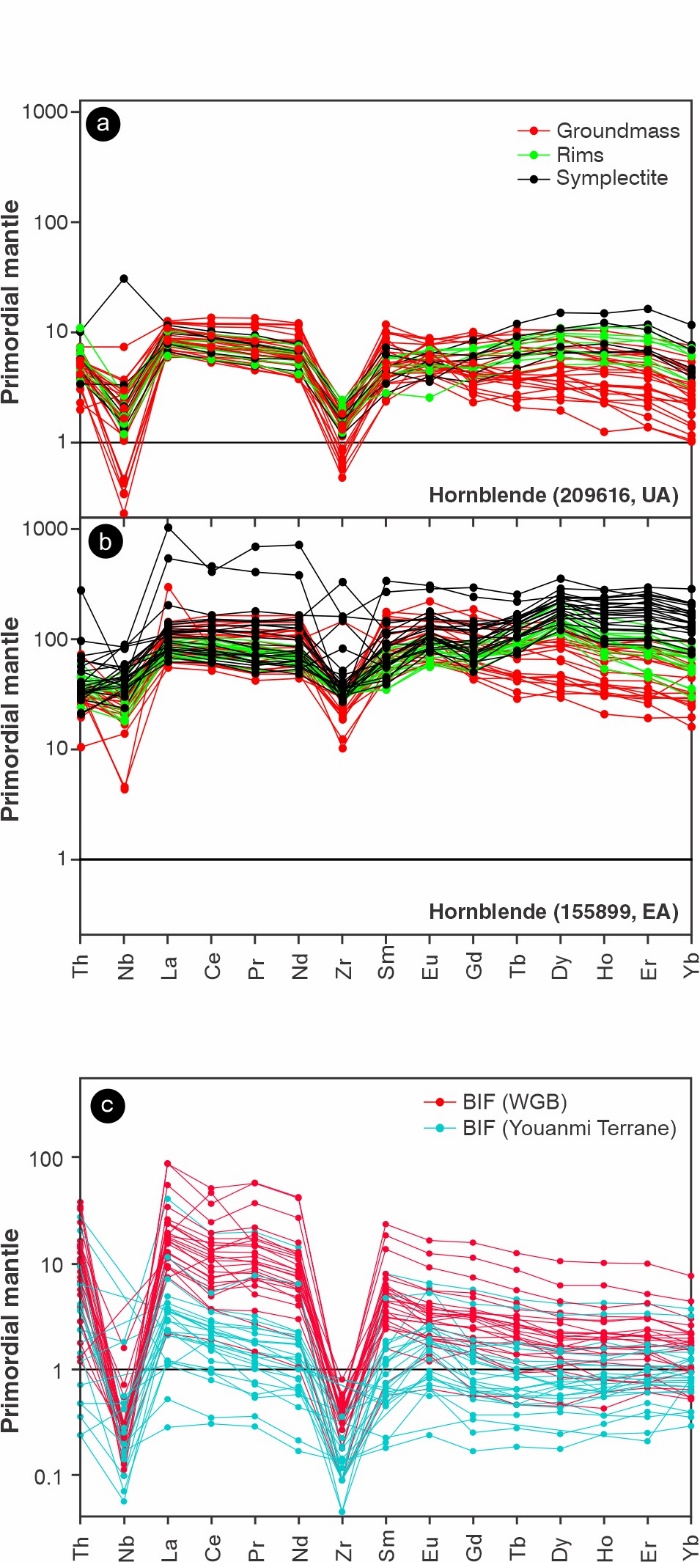


**Supplementary Figure 3.** **Trace element diagrams for garnet and amphibole in amphibolite, and for BIF.** (a) and (b) Primitive mantle normalised trace element diagrams showing compositional variation within hornblende for an unenriched (UA) and enriched (EA) amphibolite (normalization factors from^1^). Relevant analyses are shown in Supplementary Dataset 5. (c) Whole-rock incompatible trace-element patterns of BIF, normalised against concentrations in primordial mantle, and compared with Youanmi Terrane BIF, sampled away from the WGB. The corresponding analyses are shown in Supplementary Dataset 6.

[**Supplementary Dataset 2**](https://osf.io/yqrbj?view_only=118264df69a142f19c9918ce243b1d28)**.** Whole-rock major and trace element compositions of amphibolite from the WGB.

[**Supplementary Dataset 3**](https://osf.io/m9tqs?view_only=118264df69a142f19c9918ce243b1d28)**.** Whole-rock major and trace element compositions of basalts from the Yilgarn Craton.

[**Supplementary Dataset 4**](https://osf.io/59hgb?view_only=118264df69a142f19c9918ce243b1d28)**.** Laser ablation inductively coupled plasma mass spectrometry **(**LA-ICP-MS) trace element analyses of garnet.

[**Supplementary Dataset 5**](https://osf.io/2aqv5?view_only=118264df69a142f19c9918ce243b1d28)**.** LA-ICP-MS trace element analyses of hornblende.

[**Supplementary Dataset 6**](https://osf.io/5hnbk?view_only=118264df69a142f19c9918ce243b1d28). Representative bulk rock composition of BIF from the WGB, and from the rest of the Youanmi Terrane (Yilgarn Craton).


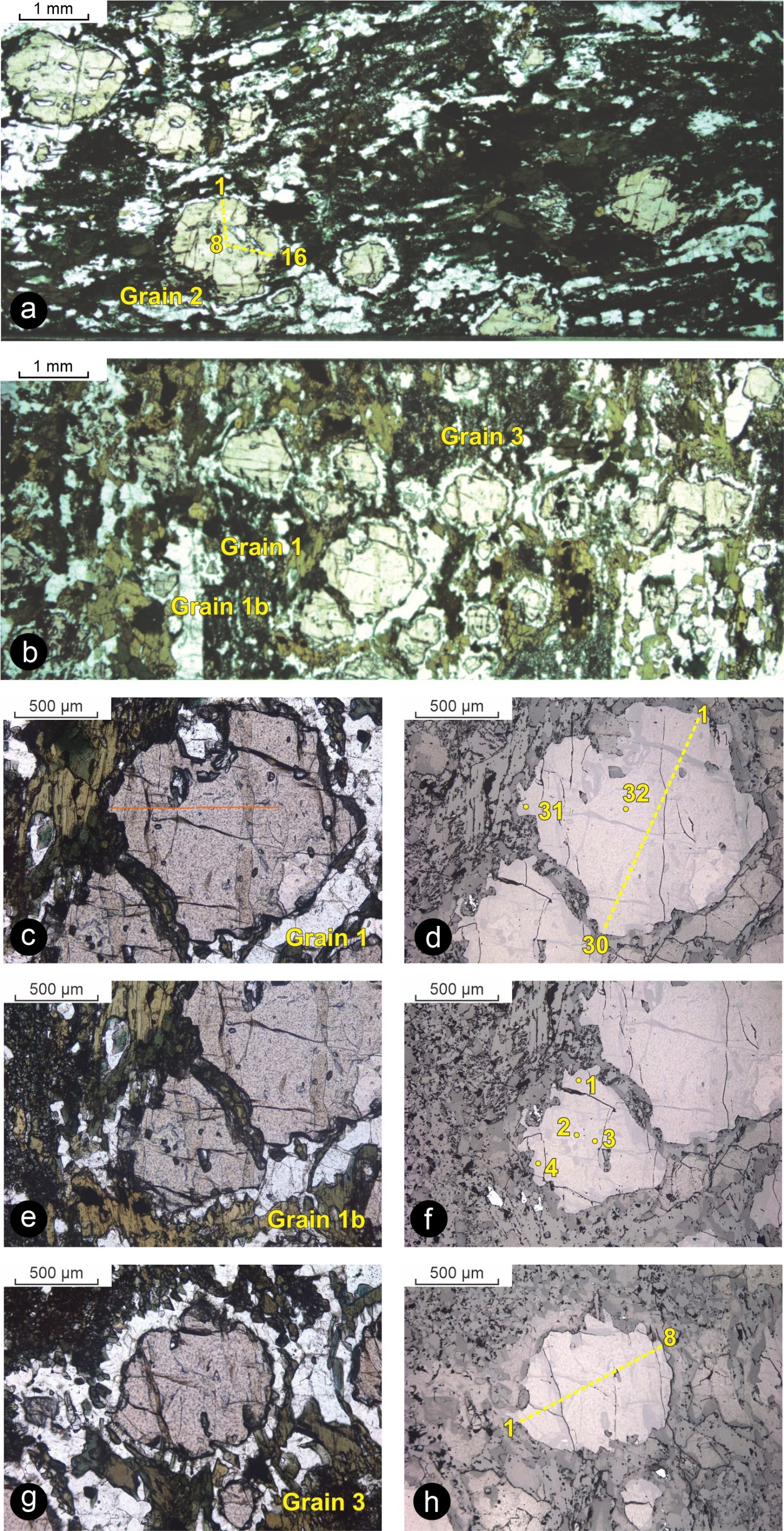


**Supplementary Figure 4.** **Thin-section maps for analysed garnet grains, unenriched amphibolite.** (a) and (b): whole-thin section micrographs of sample 214208 and images of the analysed garnet grains in transmitted (c,e,g) and reflected light (d,f,h). Dotted lines show profile across the individual grains with the staring and end point of analyses (e.g., the profile of grain 1 consists of 30 individual Secondary ion mass spectrometer (SIMS) oxygen isotope spots).


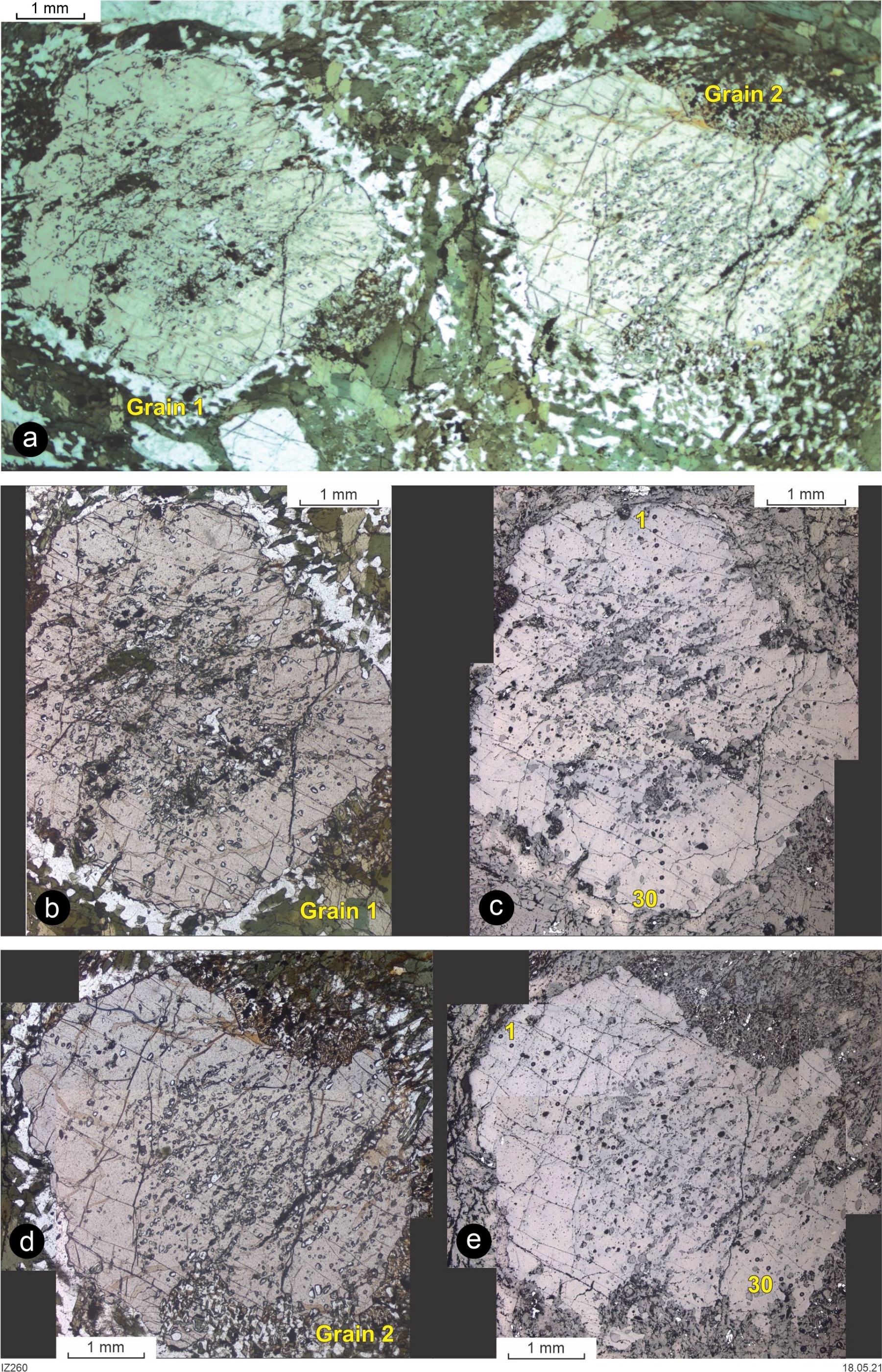


**Supplementary Figure 5.** **Thin-section maps for analysed garnet grains, enriched amphibolite.** (a) low-magnification micrographs of sample 155899, with garnet grains analysed for their oxygen isotope signatures. (b) and (c) show grain 1 in transmitted and reflected light (respectively), where dotted lines show profile across the individual grains with the starting and end point of analyses (e.g., the profile of grain 1 consists of 30 individual SIMS oxygen isotope spots). (d) and (e) show grain 2 in transmitted and reflected light (respectively) with the analytical transect indicated by the dotted line (30 analyses).

**
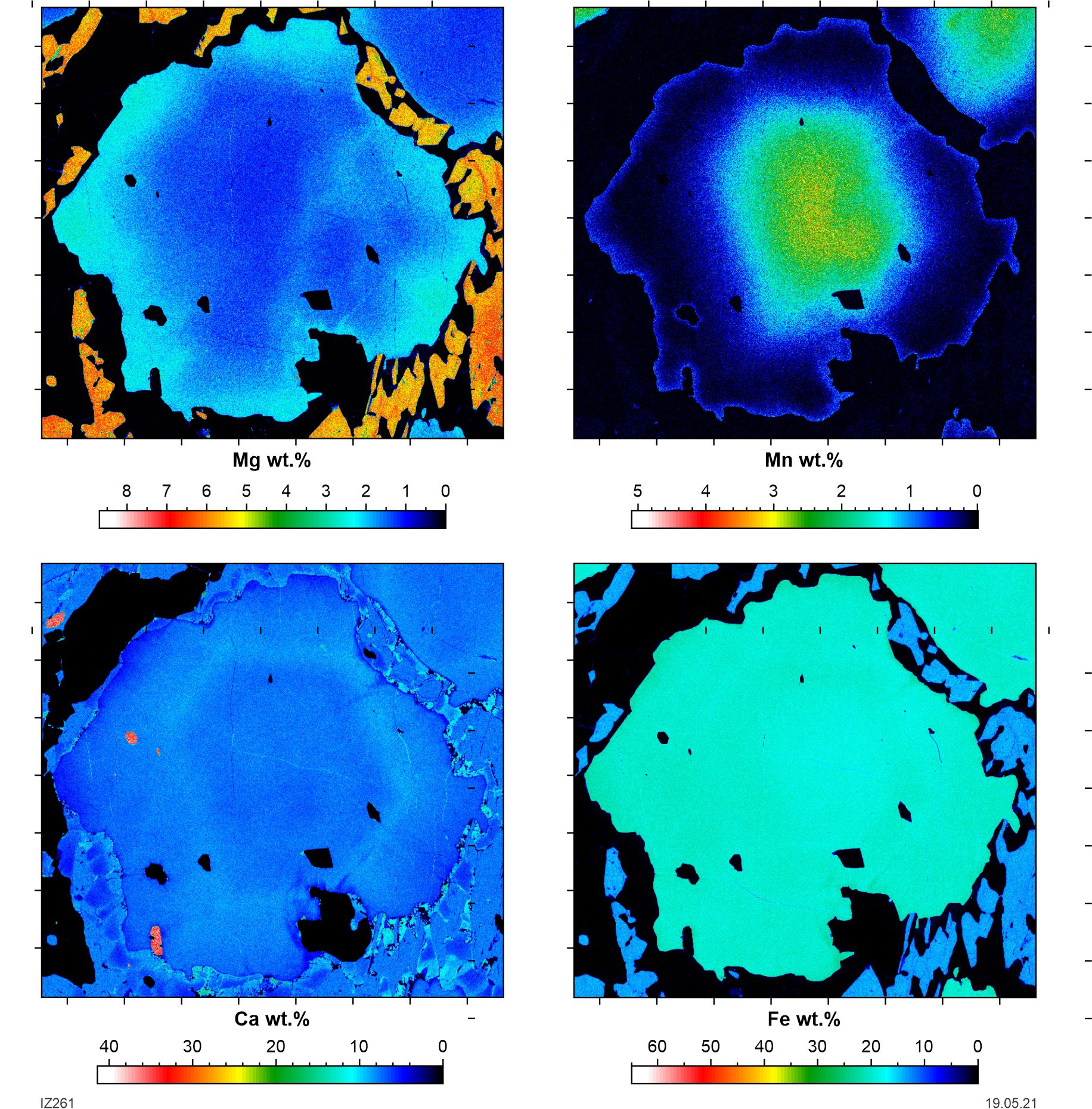
**

**Supplementary Figure 6.** **Quantitative electron probe microanalyzer (EPMA) elemental maps**. Quantitative EPMA elemental map of Mg, Mn, Ca and Fe in garnet of sample 214208 (Grain 1, see supplementary Figure 1). The garnet grains show typical zonation of major elements (e.g., Mn enrichment of core) commonly found in garnets formed during high-grade metamorphism^2^.


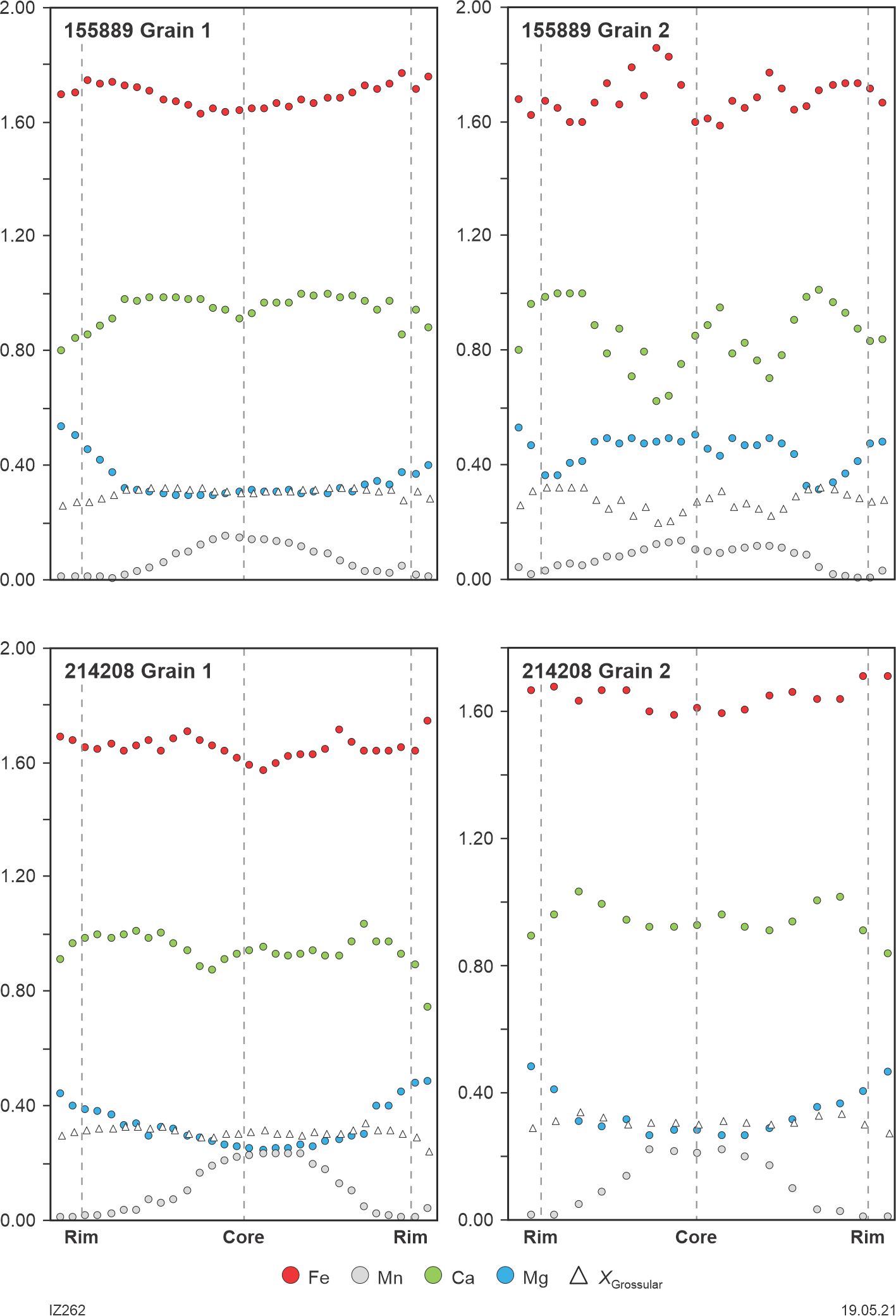


**Supplementary Figure 7. Major element contents in garner grains.** Fe, Ca, Mg, Mn contents (a.p.f.u.) of garnets based on 12 oxygen and corresponding grossular contents used for matrix correction of SIMS oxygen isotope analyses. Chemical zonation of Mn, Ca, and Mg can be attributed to the metamorphic growth of garnet^3^ and the attempted geochemical equilibration with the rock matrix during its formations.


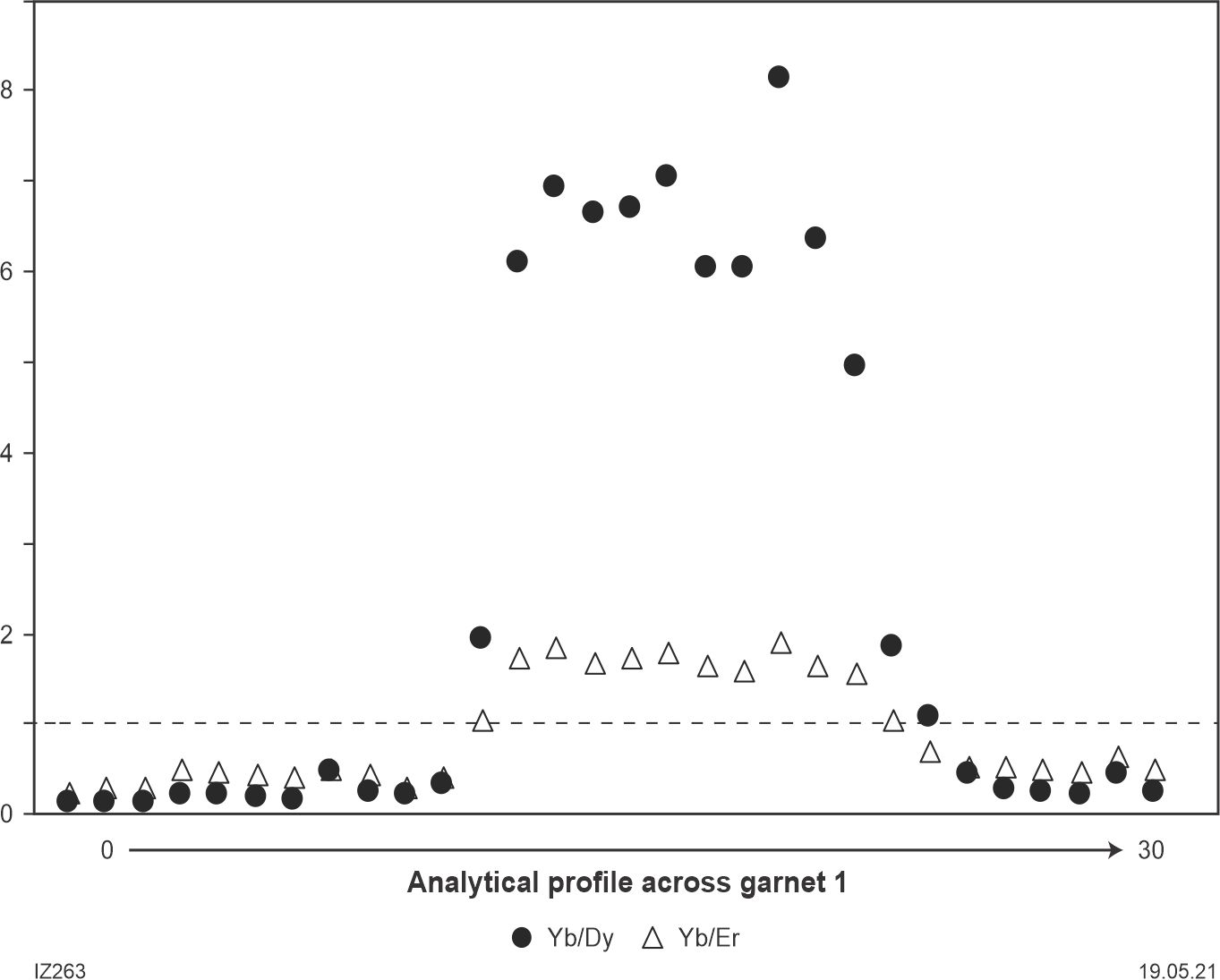


**Supplementary Figure 8.** **Yb/Dy and Yb/Er trace element ratios in garnet 1 of sample 214208.** The profile across the garnet shows Yb/Dy and Yb/Er ratios >1 in the core compared to the rim (<1). This bell-shaped pattern of the ratios between heavy rare Earth elements (HREE) and middle rare Earth elements (MREE) is likely a result of Rayleigh fractionation of REEs during crystal growth^4^.


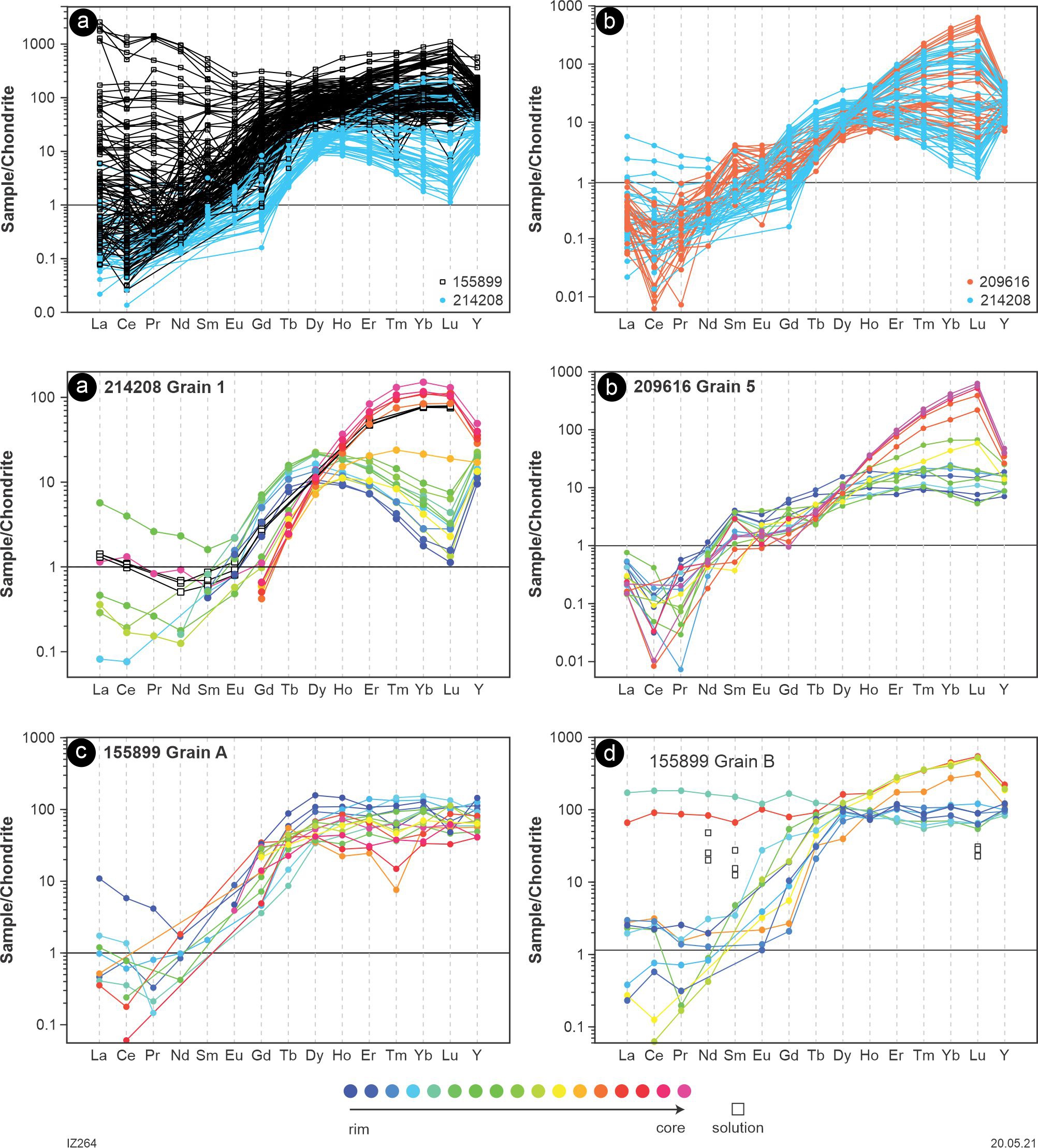


**Supplementary Figure 9.** **Trace element contents in garner grains.** (a) Comparison of trace elements signatures across a garnet grain in sample 214208. Note that the core is enriched in HREE compared to the rims. Bulk grain solution analyses of the same sample are shown as white squares. (b) The trace element pattern in garnet of sample 209616 shows a less distinct HREE depletion in rims compared to sample 214208. (c) and (d) Trace-element signatures in garnets of sample 155899 show different patterns in individual grains. Some grains do not show a difference between core and rim (c), while others show cores enriched in HREE and, in part, in light rare Earth elements (LREE) (d).

[**Supplementary Dataset 7**](https://osf.io/3rw79?view_only=118264df69a142f19c9918ce243b1d28)**.** EPMA measurements of garnet.

[**Supplementary Dataset 8**](https://osf.io/nm92f?view_only=118264df69a142f19c9918ce243b1d28)**.** LA-ICP-MS trace element analyses of garnet.

[**Supplementary Dataset 9**](https://osf.io/vmhkw?view_only=118264df69a142f19c9918ce243b1d28)**.** Raw ^18^O/^16^O ratios and the corrected δ^18^O values of SIMS analyses in garnet (quoted with respect to Vienna Standard Mean Ocean Water, or VSMOW, in per mil).

**Supplementary section 2. Zircon, monazite and garnet geochronology**

The Sm-Nd isotope data obtained from the same dissolved whole-rock and garnet fractions have a small spread of ^147^Sm/^144^Nd rations (0.16 to 0.22) and define a slope on an isochron diagram that corresponds to a date of 2535 ± 52 Ma (mean square weighted deviation, MSWD = 0.26). The young age relative to that obtained from Lu–Hf is attributed to a later thermal resetting, as is evident in some of the zircon U–Pb data from adjacent samples plotted above. The limited Sm/Nd fractionation between the whole rock and constituent garnets may reflect diffusional exchange during this resetting.

[**Supplementary Dataset 10**](https://osf.io/ensmd?view_only=118264df69a142f19c9918ce243b1d28)**.** Ion microprobe U-Th-Pb analyses of monazite and zircon.

[**Supplementary Dataset 11**](https://osf.io/762wj?view_only=118264df69a142f19c9918ce243b1d28)**.** Sm-Nd and Lu-Hf isotope data.

**Supplementary section 3. Metamorphism**

We analysed the same leucoamphibolite sample (219364, selected also for zircon geochronology and geochemistry) for detailed metamorphic analysis. We integrate these results with existing metamorphic data from the Waroonga Shear Zone^5^, to define a near-complete exhumation *P–T–t* path.

**Leucoamphibolite**

**Petrography**

Leucoamphibolite contains garnet, hornblende, plagioclase, quartz, magnetite and ilmenite, with minor apatite and zircon, and trace biotite. Leucoamphibolite is typically fine grained (1–2 mm grain size) and contains up to 40% garnet. At the hand sample scale, leucoamphibolite commonly shows a weak foliation, together with a discontinuous compositional layering defined by quartzofeldspathic lenses, garnet-rich, amphibole-rich and oxide-rich layers (Supplementary Figure 10a). In thin section, melanocratic layers are mainly composed of garnet, amphibole and magnetite porphyroblasts, within a quartzofeldspathic matrix. Garnet shows embayed grain boundaries and contains abundant rounded inclusions of quartz. Small anhedral garnet relics occur within hornblende and plagioclase. Opaque minerals comprise magnetite, in some cases with extensive hematite alteration, and ilmenite (sensu lato). The Fe–Ti oxides are commonly intergrown but also occur as discrete grains throughout the matrix (Supplementary Figure 10b). Hornblende occurs up to 1 mm in size, and is commonly in close association with garnet. Coarse-grained hornblende may have embayed grain boundaries, although rare euhedral grains up to 0.3 mm in length occur within plagioclase.

The typical microstructure shows plagioclase films coating embayed hornblende and Fe–Ti oxide grain boundaries or large, amoeboid and poikilitic plagioclase grains enclosing embayed hornblende and Fe–Ti oxide grains (Supplementary Figure 10c and d). Plagioclase occurs as poikilitic grains and as films coating grain boundaries, and is interpreted here to represent former melt^6^. As a whole, the nearly perfect preservation of microstructures typical of migmatitic rocks, together with a macroscopic weak fabric and the occurrence of polygonal quartz aggregates showing dihedral angles close to 120° (Supplementary Figure 10e), suggest that leucoamphibolite recorded little deformation during melt crystallization^6^, and negligible retrograde overprint during exhumation along the Waroonga Shear Zone.

Based on these petrographic observations, the peak metamorphic assemblage in sample 219364 is interpreted to contain garnet, ilmenite, quartz, plagioclase and melt. Hornblende is interpreted to post-date garnet, and is inferred to have grown in the presence of melt, but at slightly lower *P–T* conditions than peak metamorphism.


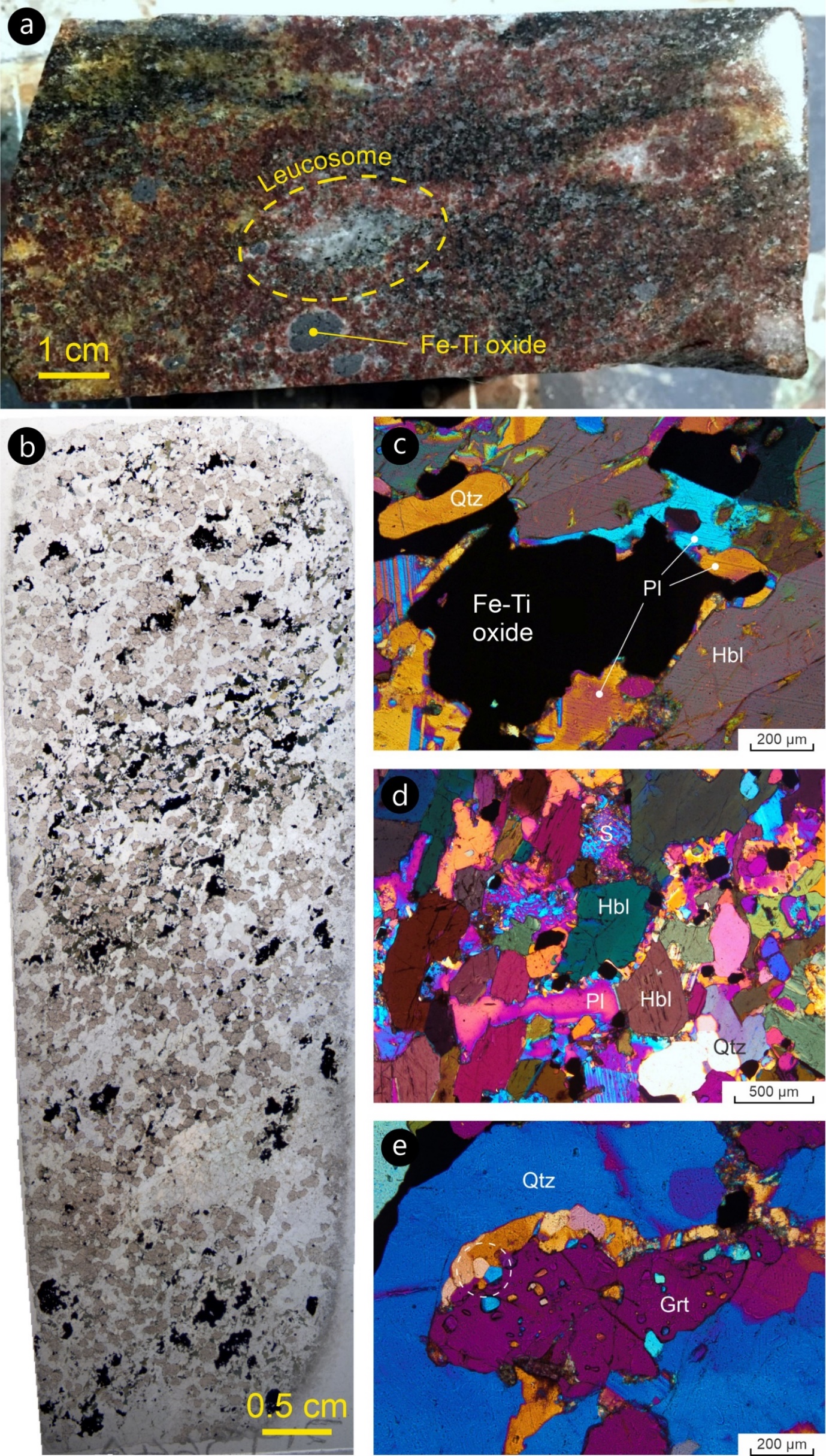


**Supplementary Figure 10.** **Microstructure of leucotonalite.** (a) Representative hand-sample scale compositional layering in leucoamphibolite (219364). (b) Whole-thin section photomicrograph of 219364, showing discontinuous compositional layering defined by quartz-rich layers alternating with garnet–amphibole–magnetite-rich layers. Plane polarized light. The long side of the image is 7.5 cm-long. (c) Garnet–amphibole–magnetite melanocratic layer. The phases in blue and yellow consist of single, optically continuous plagioclase grains coating the magnetite grain. See (a) for comparison. Crossed polars, gypsum plate inserted. (d) Amphibole–magnetite-rich layer. Phases in blue and yellow represent anhedral plagioclase grains, interpreted as former melt-filled pore structure between amphibole and magnetite. Crossed polars, gypsum plate inserted. (e) Garnet porphyroblast within quartzofeldspathic matrix. In the centre-left portion of the image, quartz aggregates show polygonal aggregates of nearly isometric and strain-free grains, with dihedral angles close to 120°, a feature that is indicative of as typical of static crystallization from melt^6^.

**Mineral chemistry**

Representative mineral compositions are provided in Supplementary Dataset 12; mineral abbreviations after^7^. Garnet porphyroblasts are almandine-rich, with x(Grt) [= Fe^2+^ ⁄(Fe^2+^ + Mg + Ca)] values of 0.87–0.90. Garnet grains have z(Grt) [= Ca ⁄(Fe^2+^ + Mg + Ca)] contents ranging from 0.21 to 0.29, although most analyses have values between 0.23 and 0.26. Porphyroblasts show very little compositional zoning, although the very outer rim typically contains higher z(Grt) contents (up to 0.28–0.29). Small garnet relics in hornblende have the lowest z(Grt) values, whereas garnet relics in plagioclase typically have z(Grt) values between 0.24 and 0.26. Plagioclase shows a range of compositions, with ca(Pl) [= Ca/(Ca + Na)] values typically between 0.66 and 0.76. Some grains show heterogeneous alteration with relic domains preserving ca(Pl) values as low as 0.11; these grains are interpreted as altered primary grains. Hornblende shows little compositional variation within individual grains, although there are slight differences between different petrographic settings, with x(Hbl) [= Fe2+ ⁄(Fe2+ + Mg)] values from 0.53 to 0.63, and y(Hbl) [= Al on M_2_ site] contents between 0.24 and 0.35. Small euhedral grains within plagioclase have x(Hbl) = 0.55–0.58 and y(Hbl) = 0.24–0.35.

**Phase equilibria modelling**

We investigated the metamorphic evolution of sample 219364 by using phase equilibria modelling, based on the bulk rock composition (Supplementary Dataset 13). The sample contains appreciable apatite, which was removed from the effective bulk composition. Thermodynamic calculations were performed in the 10-component NCKFMASHTO (Na_2_O–CaO–K_2_O–FeO–MgO–Al_2_O_3_–SiO_2_–H_2_O–TiO_2_–O_2_) system using THERMOCALC version tc3409 and the internally consistent thermodynamic dataset of 10. The following a–x relations were used in the modelling: metabasite melt, augite, and hornblende^8^; garnet, orthopyroxene, biotite, chlorite, and muscovite–paragonite^9^; olivine and epidote^10^; magnetite–spinel^3^; ilmenite–hematite^11^; and plagioclase and K-feldspar^12^. Pure phases included quartz, rutile, sphene, and aqueous fluid (H_2_O). Compositional and modal isopleths for all phases were calculated using TCInvestigator^13^.

Based on the LOI value from this sample (~0 wt.%; Supplementary Dataset 13), the composition is anhydrous. However, the predicted assemblages using this anhydrous composition are inconsistent with the observed equilibria; in particular, predicted hornblende modes are underestimated as compared to observed modes of ~ 15%. In order to produce hornblende contents that more closely approximate the assemblage preserved in the sample, the H_2_O content was adjusted so that the solidus was just H_2_O-saturated at pressures above 5 kbar. The O content was selected based on the Fe_2_O_3_:FeO proportion determined by Fe^2+^ titration, which reproduces the observed assemblage.

The *P–T* pseudosection for sample 219364 was constructed over a temperature range of 620–850 °C and between pressures of 3 and 12 kbar (Supplementary Figure 11). Pressures above 12 kbar were not modelled as it is beyond the calibration of the mafic melt model^8^. The solidus is predicted to have a maximum temperature of 760 °C at 4 kbar, extending to lower temperatures at higher and lower pressures. As described above, the solidus is H_2_O-saturated above 5 kbar. Garnet is stable above 3.5 kbar across the range of modelled conditions. Coexisting plagioclase + anorthite-bearing assemblages are predicted at pressures below 8 kbar. Epidote-bearing assemblages are stable above 7.5 kbar under subsolidus conditions, with the lower pressure limit extending to higher pressures with increasing temperature. Compositional and modal isopleths for selected phases are shown on Supplementary Figure 12 and 13, respectively.

The inferred peak metamorphic assemblage for sample 219364 comprises garnet, quartz, plagioclase, ilmenite, and melt, which corresponds to the Grt–Liq (+ Qz + Pl + Ilm) field that is stable over a wide *P–T* range above 680 °C and 7.5 kbar (‘1’ on Supplementary Figure 11). Garnet compositions within this broad field have z(Grt) values of 0.24 (Supplementary Figure 12), which are the same as observed values. Decompression (with or without cooling) into the Grt–Mag–Liq (+ Qz + Pl + Ilm) field accounts for the growth of magnetite (‘2’ on Supplementary Figure 11). This segment of the retrograde path corresponds to an increase in plagioclase mode at the expense of garnet (Supplementary Figure 12), which may account for plagioclase rimming resorbed garnet and garnet relics within plagioclase. Predicted plagioclase compositions along the decompression path range from 0.60 to 0.70 (Supplementary Figure 12), broadly consistent with observed ca(Pl) values. Hornblende growth at the expense of garnet, melt and plagioclase is predicted with further cooling into the Grt–Hbl–An–Mag–Liq (+ Qz + Pl + Ilm) field (‘3’ on Supplementary Figure 11). Additional cooling passes into the narrow Grt–Hbl–Bt–An–Mag–Liq (+ Qz + Pl + Ilm), followed by crystallization at the solidus. Passing through this narrow field results in distinct modal changes in hornblende and plagioclase (Supplementary Figure 13), alternating between consumption and growth over a very small temperature range. Assuming favorable kinetics in the presence of melt, these reactions may explain the occurrence of both embayed and euhedral hornblende associated with plagioclase.

The preserved assemblage in the sample is garnet–hornblende–magnetite–quartz–plagioclase–ilmenite, which records the conditions of crystallization at the solidus. These conditions are inferred to correspond to the Grt–Hbl–Bt–An–Mag–H2O (+ Qz + Pl + Ilm) field, which is stable between 640–710 °C and 5–7.5 kbar (‘4’ on Supplementary Figure 11). Up to 12 mol.% anorthite is predicted in this field at pressures near 5 kbar, with the modal abundance decreasing with increasing pressure. At 7 kbar, 2 mol.% anorthite is predicted (Supplementary Figure 13). Although anorthite has not been identified in thin section, its presence cannot be ruled out, especially in fine-grained myrmekitic intergrowths (Supplementary Figure 10d). We observed rare biotite is in thin section, which likely accounts for the 2 mol.% value (at the solidus) predicted in the model.

In summary, estimates for peak metamorphism are in excess of 680 °C and 7.5 kbar, but likely did not reach much higher than 850 °C. At temperatures above 850 °C, predicted melt volumes are in fact >10 mol.% (~ vol.%; Supplementary Figure 13), in contradiction with the lack of geochemical evidence for melt loss in leucoamphibolite.

Field relationships, together with geochemical and isotopic data, indicate that all the greenstone lithologies of the Waroonga Greenstone Belt experienced the same tectonometamorphic evolution. Therefore, although peak pressures for sample 219364 are more poorly constrained, peak *P–T* estimates of 11.2–13 kbar and 765–850 °C recorded by amphibolite (sample 209029), must have been recorded by the Waroonga Greenstone Belt as a whole. Following peak metamorphism, microstructural observations and mineral chemistry from sample 219364 support a post-peak history of decompression and cooling to conditions of melt crystallization at ~700 °C and 5–7 kbar.


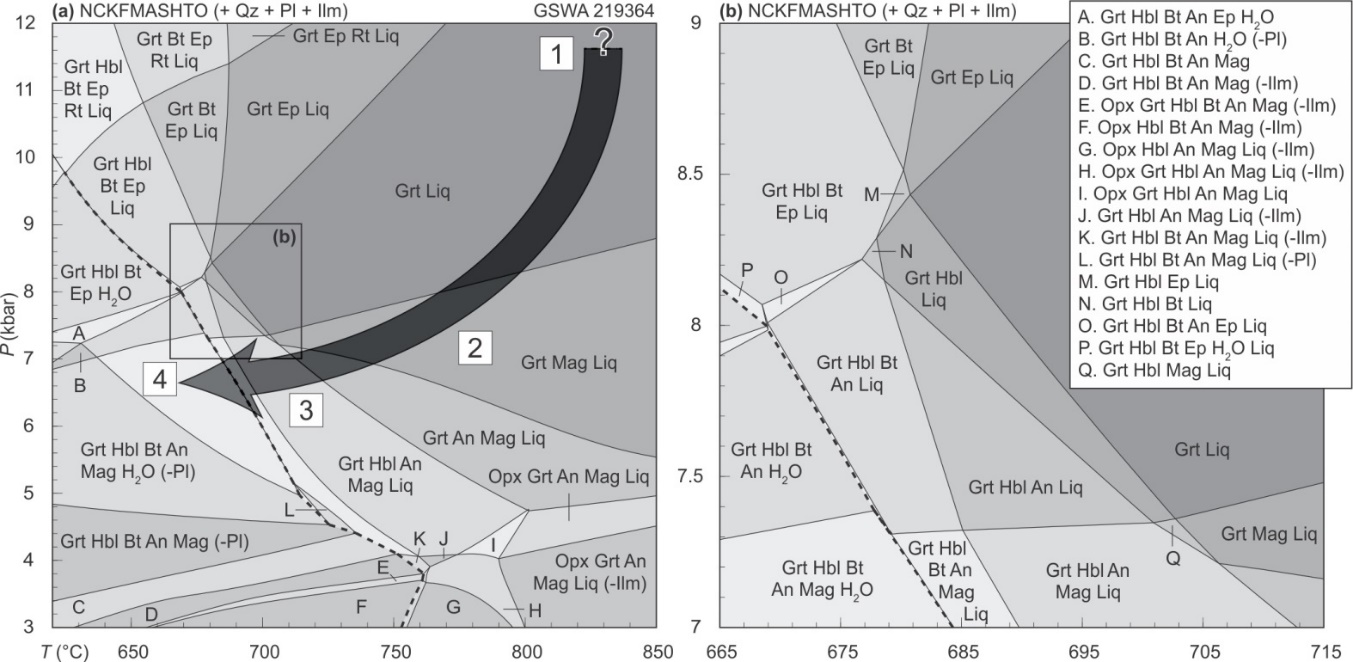


**Supplementary Figure 11.** **Pseudosection for sample 219364**. (a) *P–T* pseudosection calculated for sample 219364. Schematic *P–T* path is represented by the grey arrow; steps ‘1’ to ‘4’ are described in the text. Dashed line corresponds to the solidus. (b) Expanded *P–T* range for the central part of the pseudosection.


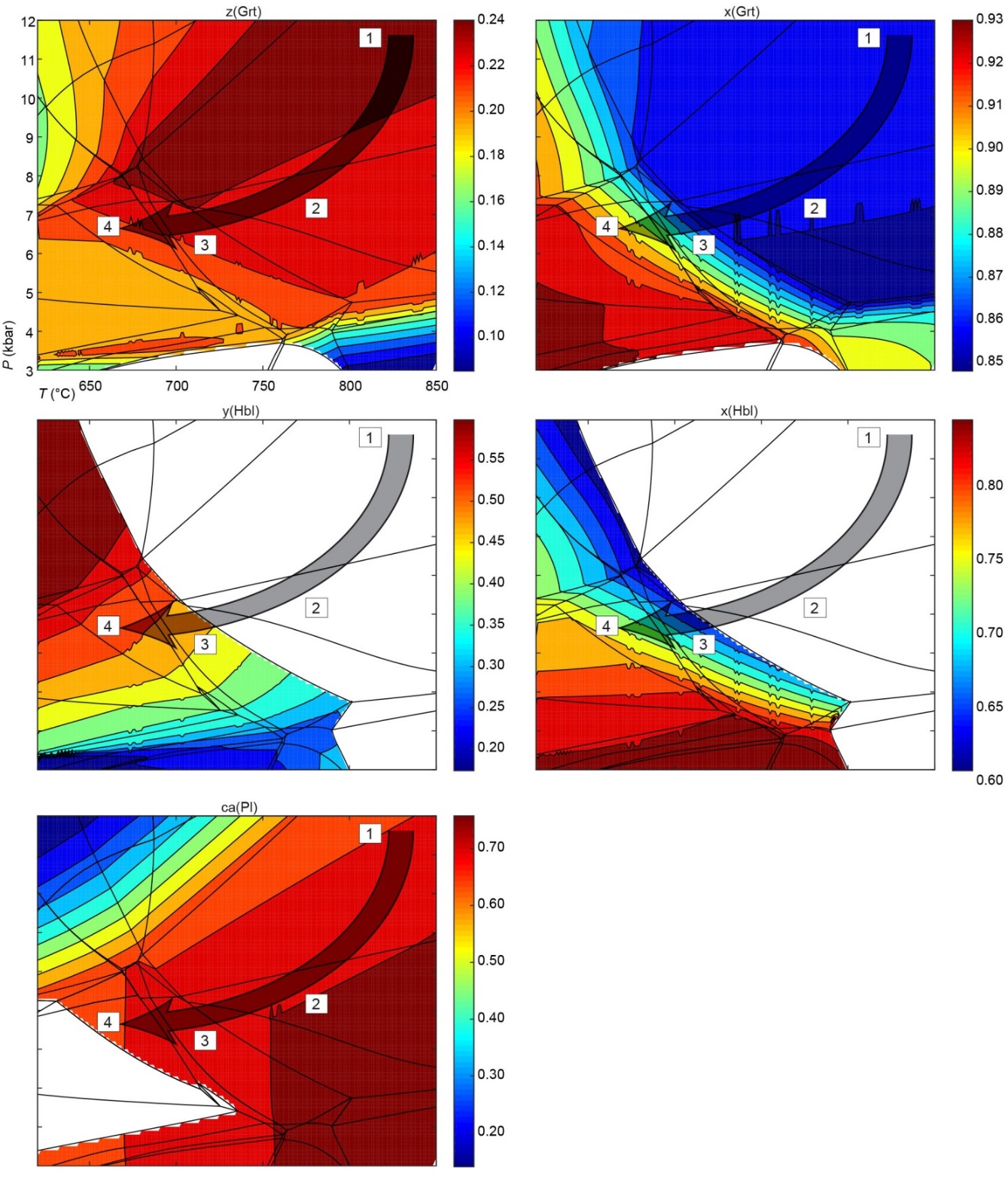


**Supplementary Figure 12.** **Compositional isopleths for garnet, hornblende and plagioclase**. Compositional isopleths for garnet, hornblende and plagioclase from Supplementary Figure 11: z(Grt) [= Ca ⁄(Fe2+ + Mg + Ca)]; x(Grt) [= Fe2+ ⁄(Fe2+ + Mg + Ca)]; y(Hbl) [= Al on M2 site]; x(Hbl) [= Fe2+ ⁄(Fe2+ + Mg)]; ca(Pl) [= Ca/(Ca + Na)].


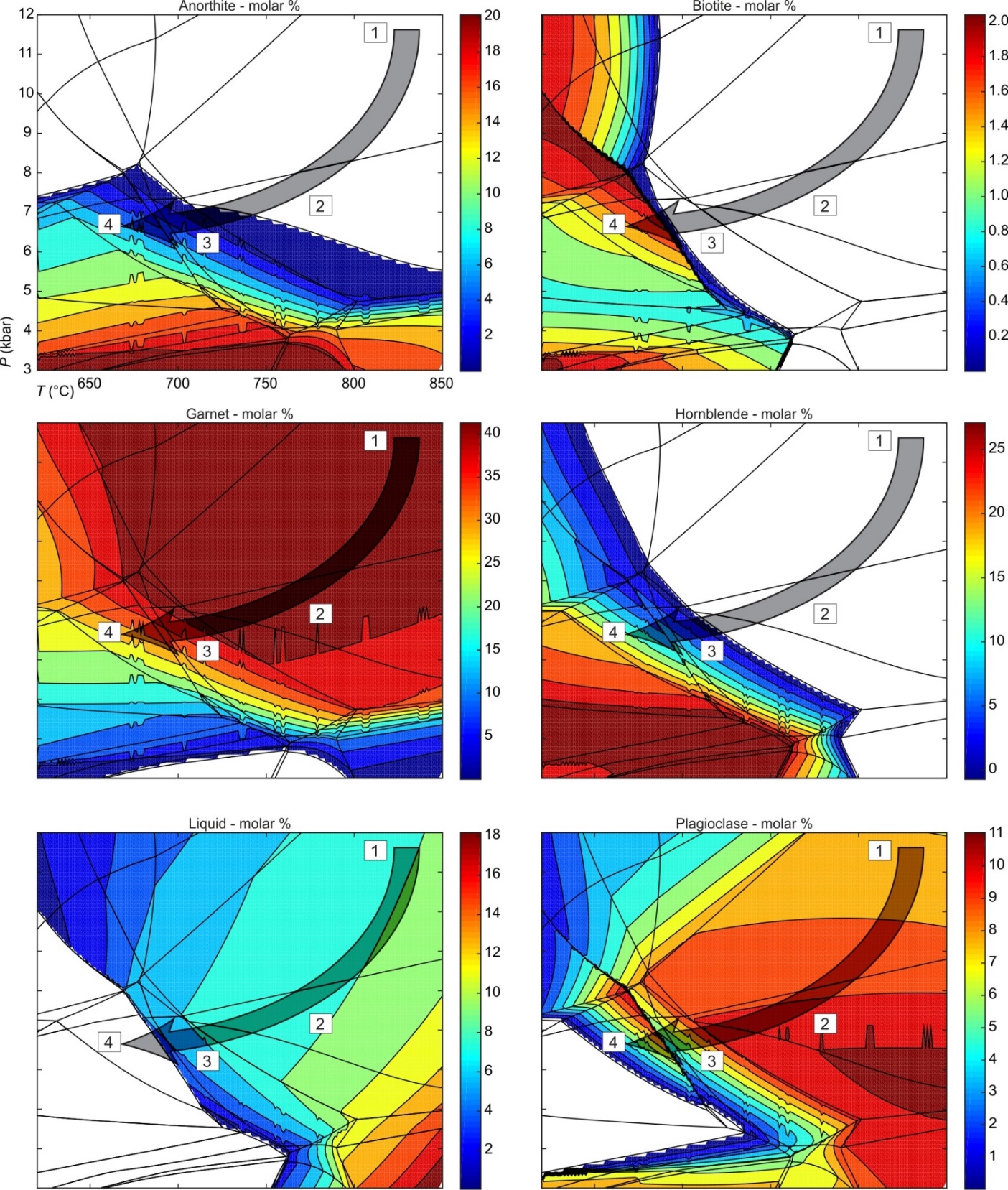


**Supplementary Figure 13.** Modal isopleths (mol.%; approximately equal to vol.%) for key phases from Supplementary Figure 12.

The metamorphic conditions recorded by the WGB yield apparent thermal gradients of 70 °C/kbar at c. 2730 Ma, increasing to 90 °C/kbar at 2717–2700 Ma, with the highest thermal gradients of 180 °C/kbar at c. 2660 Ma. These values respectively fall within intermediate dT/dP (37.5–77.5 °C/kbar) and high dT/dP (> 775 °C/kbar) styles of metamorphism that are widely registered in the rock record back to c. 2.8 Ga^14^ (Supplementary Figure 14). The intermediate dT/dP metamorphism at c. 2730 Ma is consistent with generally thinner lithosphere during the Archean^15^, whereas the progressive increase to high dT/dP metamorphism by c. 2660 Ma is clearly influenced by the emplacement of voluminous syntectonic granites (Waroonga gneiss) along the Waroonga shear zone, and thermal perturbations within the crust at this time.

**
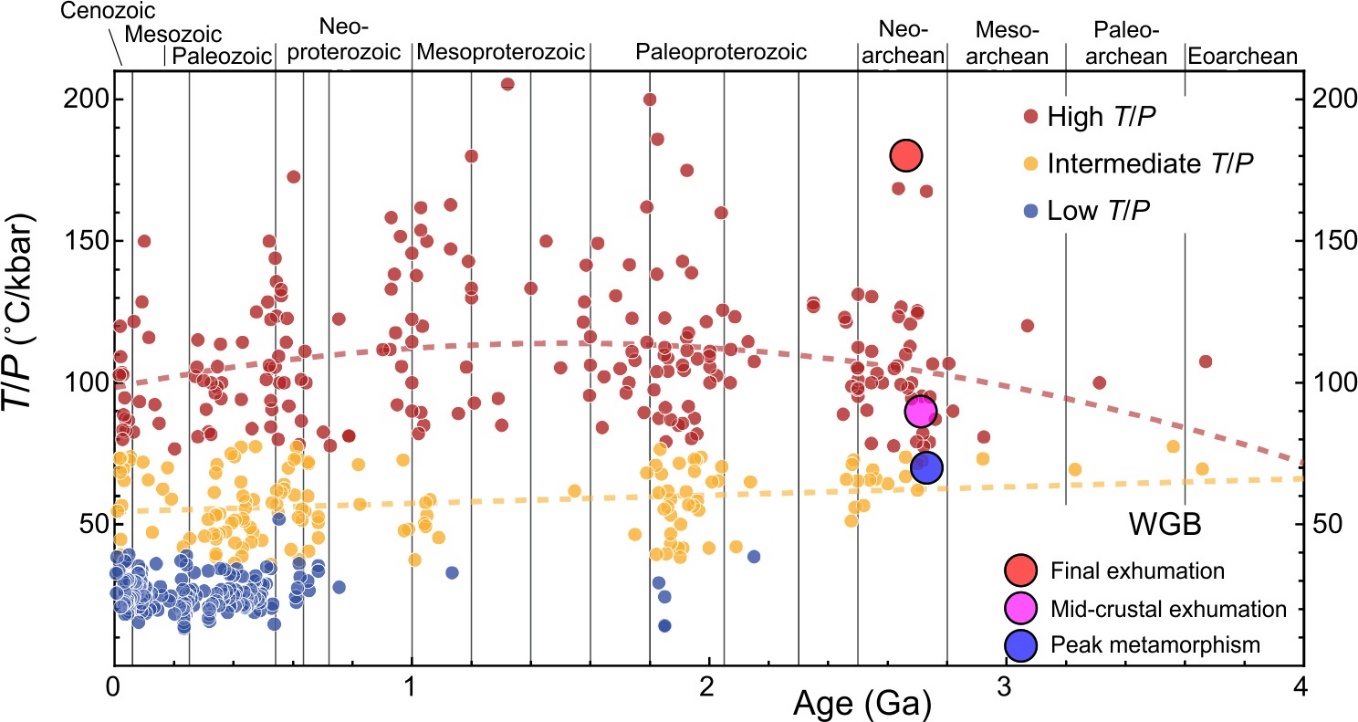
**

**Supplementary Figure 14**. **Metamorphic thermobaric ratios through time**. *P–T* data calculated for the three main metamorphic steps recorded by the Waroonga Greenstone Belt (compare with Fig. 7), plotted in the metamorphic thermobaric ratios [temperature (T), pressure (P) (T/P)] for 564 localities^15^. The dashed lines show a second-order polynomial regression of the data for the high T/P (red) and a linear regression of the data for the intermediate T/P (orange) types, respectively. Developed after^15^.

[**Supplementary Dataset 12**](https://osf.io/vapx7?view_only=118264df69a142f19c9918ce243b1d28). Representative mineral compositions for sample 219364.

[**Supplementary Dataset 13**](https://osf.io/dfw5u?view_only=118264df69a142f19c9918ce243b1d28)**.** Compositions used in phase equilibria modelling for sample 219364.

**Supplementary References**

1. Sun, S.-S. & McDonough, W. Chemical and isotopic systematics of oceanic basalts: implications for mantle composition and processes. *Geological Society, London, Special Publications* **42**, 313–345 (1989).

2. Caddick, M. J., Konopásek, J. & Thompson, A. B. Preservation of Garnet Growth Zoning and the Duration of Prograde Metamorphism. *Journal of Petrology* **51**, 2327–2347 (2010).

3. White, R. W., Powell, R. & Clarke, G. L. The interpretation of reaction textures in Fe-rich metapelitic granulites of the Musgrave Block, central Australia: constraints from mineral equilibria calculations in the system K2O-FeO-MgO-Al2O3-SiO2-H2O-TiO2-Fe2O3. *Journal of Metamorphic Geology* **20**, 41–55 (2002).

4. Otamendi, J. E., de la Rosa, J. D., Patiño Douce, A. E. & Castro, A. Rayleigh fractionation of heavy rare earths and yttrium during metamorphic garnet growth. *Geology* **30**, 159 (2002).

5. Zibra, I. *et al.* On thrusting, regional unconformities and exhumation of high-grade greenstones in Neoarchean orogens. The case of the Waroonga Shear Zone, Yilgarn Craton. *Tectonophysics* **712–713**, 362–395 (2017).

6. Holness, M. B., Cesare, B. & Sawyer, E. W. Melted rocks under the microscope: Microstructures and their interpretation. *Elements* **7**, 247–252 (2011).

7. Whitney, D. L. & Evans, B. W. Abbreviations for names of rock-forming minerals. *American Mineralogist* **95**, 185–187 (2010).

8. Green, E. C. R. *et al.* Activity-composition relations for the calculation of partial melting equilibria in metabasic rocks. *Journal of Metamorphic Geology* **34**, 845–869 (2016).

9. White, R. W., Powell, R., Holland, T. J. B., Johnson, T. E. & Green, E. C. R. New mineral activity-composition relations for thermodynamic calculations in metapelitic systems. *Journal of Metamorphic Geology* **32**, 261–286 (2014).

10. Holland, T. J. B. & Powell, R. An improved and extended internally consistent thermodynamic dataset for phases of petrological interest, involving a new equation of state for solids. *Journal of Metamorphic Geology* **29**, 333–383 (2011).

11. White, Powell, Holland & Worley. The effect of TiO2 and Fe2O3 on metapelitic assemblages at greenschist and amphibolite facies conditions: mineral equilibria calculations in the system K2O-FeO-MgO-Al2O3-SiO2-H2O-TiO2 -Fe2O3. *Journal of Metamorphic Geology* **18**, 497–511 (2000).

12. Holland, T. & Powell, R. Activity?composition relations for phases in petrological calculations: an asymmetric multicomponent formulation. *Contributions to Mineralogy and Petrology* **145**, 492–501 (2003).

13. Pearce, M. A., White, A. J. R. & Gazley, M. F. TCInvestigator: automated calculation of mineral mode and composition contours for <scp>thermocalc</scp> pseudosections. *Journal of Metamorphic Geology* **33**, 413–425 (2015).

14. Brown, M. & Johnson, T. Secular change in metamorphism and the onset of global plate tectonics. *American Mineralogist* **103**, 181–196 (2018).

15. Brown, M., Johnson, T. & Gardiner, N. J. Plate Tectonics and the Archean Earth. *Annu Rev Earth Planet Sci* **48**, (2020).
