# Supplementary material for: Greenstone burial–exhumation cycles at the late Archean transition to plate tectonics
Source: Nat Commun. 2022 Dec 22;13:7893. doi: 10.1038/s41467-022-35208-2 (PMC9780361; doi:10.1038/s41467-022-35208-2)
Supplement: Supplementary file 3 — Supplementary Data [file 41467_2022_35208_MOESM3_ESM.pdf]

Supplementary Data 2. Whole-rock major and trace element compositions of amphibolite from the WGB.

Supplementary Data 3. Whole-rock major and trace element compositions of basalts from the Yilgarn Craton.

Supplementary Data 4. Laser ablation inductively coupled plasma mass spectrometry (LA-ICP-MS) trace element analyses of garnet.

Supplementary Data 5. LA-ICP-MS trace element analyses of hornblende.

Supplementary Data 6. Representative bulk rock composition of BIF from the WGB, and from the rest of the Youanmi Terrane (Yilgarn Craton).

Supplementary Data 7. EPMA measurements of garnets.

Supplementary Data 8. LA-ICP-MS trace element analyses of garnets.

Supplementary Data 9. Raw 18O/16O ratios and the corrected δ18O values of SIMS analyses in garnet (quoted with respect to Vienna Standard Mean Ocean Water, or VSMOW, in per mil).

Supplementary Data 10. Ion microprobe U-Th-Pb analyses of monazite and zircon.

Supplementary Data 11. Sm-Nd and Lu-Hf isotope data.

Supplementary Data 12. Representative mineral compositions for sample 219364.

Supplementary Data 13. Compositions used in phase equilibria modelling for sample 219364
